# Glucose-6-phosphate dehydrogenase is critical for suppression of cardiac hypertrophy by H_2_S

**DOI:** 10.1038/s41420-017-0010-9

**Published:** 2018-02-01

**Authors:** Aastha Chhabra, Shalini Mishra, Gaurav Kumar, Asheesh Gupta, Gaurav Kumar Keshri, Brij Bharti, Ram Niwas Meena, Amit Kumar Prabhakar, Dinesh Kumar Singh, Kalpana Bhargava, Manish Sharma

**Affiliations:** 1 Peptide and Proteomics Division, Defence Institute of Physiology and Allied Sciences (DIPAS), Delhi, India; 2 Biochemical Pharmacology Division, Defence Institute of Physiology and Allied Sciences (DIPAS), DRDO, Delhi, India; 3 Indian Institute of Toxicology Research, Uttar Pradesh, India

## Abstract

Hydrogen Sulfide (H_2_S), recently identified as the third endogenously produced gaseous messenger, is a promising therapeutic prospect for multiple cardio-pathological states, including myocardial hypertrophy. The molecular niche of H_2_S in normal or diseased cardiac cells is, however, sparsely understood. Here, we show that β-adrenergic receptor (β-AR) overstimulation, known to produce hypertrophic effects in cardiomyocytes, rapidly decreased endogenous H_2_S levels. The preservation of intracellular H_2_S levels under these conditions strongly suppressed hypertrophic responses to adrenergic overstimulation, thus suggesting its intrinsic role in this process. Interestingly, unbiased global transcriptome sequencing analysis revealed an integrated metabolic circuitry, centrally linked by NADPH homeostasis, as the direct target of intracellular H_2_S augmentation. Within these gene networks, glucose-6-phosphate dehydrogenase (G6PD), the first and rate-limiting enzyme (producing NADPH) in pentose phosphate pathway, emerged as the critical node regulating cellular effects of H_2_S. Utilizing both cellular and animal model systems, we show that H_2_S-induced elevated G6PD activity is critical for the suppression of cardiac hypertrophy in response to adrenergic overstimulation. We also describe experimental evidences suggesting multiple processes/pathways involved in regulation of G6PD activity, sustained over extended duration of time, in response to endogenous H_2_S augmentation. Our data, thus, revealed H_2_S as a critical endogenous regulator of cardiac metabolic circuitry, and also mechanistic basis for its anti-hypertrophic effects.

## Introduction

The hypertrophic growth of myocardium—conventionally thought to be a benign, compensatory response to increased cardiac workload—is increasingly being categorized as a pathological state warranting timely and effective therapeutic intervention^1,2^. It is associated with multiple cardio-vascular diseases and is a robust prognostic marker of risk for chronic heart failure^3^. Not surprisingly, therefore, numerous therapeutic targets/molecules with potential anti-hypertrophy effects have been identified^1^ and are being studied for their mechanistic basis. Some recent studies (from both animal as well as human studies) have, interestingly, revealed a strong association between multiple cardio-pathological states and hydrogen sulfide (H_2_S)—a gaseous messenger endogenously produced through elaborate enzyme systems in mammalian cells^4^. Amongst some notable evidences, the circulating and myocardial levels of H_2_S were observed to be significantly (nearly 60%) lower in animals subjected to transverse aortic constriction—culminating in pressure overload, hypertrophy and heart failure^5^. Similar observations have independently been made for isoproterenol-induced myocardial injury^6,7^, adriamycin-induced cardiomyopathy^8^ and spontaneously hypertensive rats^9^. Importantly, significantly diminished levels of circulating H_2_S have been observed in heart failure patients as well^10^, supporting general relevance of this phenomenon during cardiac dysfunction. Interestingly, exogenous donors or strategies (employing genetic tools) to augment endogenous levels of H_2_S appear to manifest ‘‘cardio-protective’’ effects during multiple pathological conditions affecting heart^5,10^.

Despite such compelling evidences, and gradual appraisal of H_2_S as an authentic signaling molecule^11^, the cellular niche of this key messenger in normal or diseased myocardium remains incompletely understood. Unlike areas such as hypoxia sensing^12,13^, there is limited information from cardiac cells describing endogenous functions of H_2_S in basal or pathological signaling pathways. It is tempting to speculate that cue-dependent downregulation of endogenous H_2_S could per se propel specific signaling pathways and culminate in cardio-pathological effects—a possible reason why therapeutic modulation of endogenous H_2_S produces distinctive cardio-protective effects. This proposition draws support from certain known effects of H_2_S in cardiovascular system, including preservation of mitochondrial function^14^, regulation of Nrf-2 signaling cascade^15^ and endothelial nitric oxide synthase (eNOS) activity^16^ (through sulfhydration of its specific cysteine residues). Furthermore, there exist strong reasons to surmise a much more intricate regulatory cascade, in addition to molecules/processes described above, regulated by H_2_S in cardiac cells. H_2_S can potentially modify, directly (post-translational), a significant number of proteins in the cells^17–20^—many of which are key regulatory molecules. Interestingly, a recent study showed that it is capable of covalently modifying several electrophilic species (such as 8-nitro cGMP) centrally involved in cellular ‘‘redox signaling’’^21^ and thus, capable of fine-tuning signal transduction in response to vivid extra-cellular or intracellular cues. Finally, the very fact that mammalian cells, including cardiomyocytes, have evolved multiple (organelle-specific) intrinsic H_2_S-generating enzyme systems/mechanisms^22^ attests colossal and larger intracellular niche for this gaseous messenger in myocardium.

We here show that adrenergic overstimulation—known to culminate in hypertrophy—rapidly downregulates endogenous H_2_S levels in cardiomyocytes. The utilization of an exogenous H_2_S donor, prior to adrenergic stimulation, prevented this effect and also, strongly counteracted hypertrophic progression, suggesting an intrinsic role of H_2_S during adrenergic stress. We, further, sought to understand global cellular networks regulated by H_2_S in cardiac cells, utilizing transcriptome sequencing and subsequently, investigated their biological significance during pathological conditions. We observed that the augmentation of endogenous H_2_S levels culminated in modulation of a significant number of genes, which composed an integrated metabolic circuitry regulating production (pentose phosphate pathway, PPP) and utilization (glutathione biosynthesis, cholesterol synthesis and NOX pathways) of cellular reducing equivalents (NADPH). Amongst such gene networks, glucose-6-phosphate dehydrogenase (G6PD)—the first and rate-limiting enzyme in PPP—emerged as a critical node (highest degree) and thus, suggesting its biological significance for effects produced by H_2_S. We present multiple experimental evidences to support such functional relevance of G6PD during H_2_S-induced suppression of cardiac hypertrophy (induced by adrenergic overstimulation) in both cellular as well as animal model systems. Our results, thus, revealed a critical role of H_2_S in regulating cardiac cell metabolism and molecular basis for its cardioprotective effects.

## Results

### Adrenergic stimulation rapidly decreases endogenous H_2_S in cardiomyocytes

**Fig. 1:**
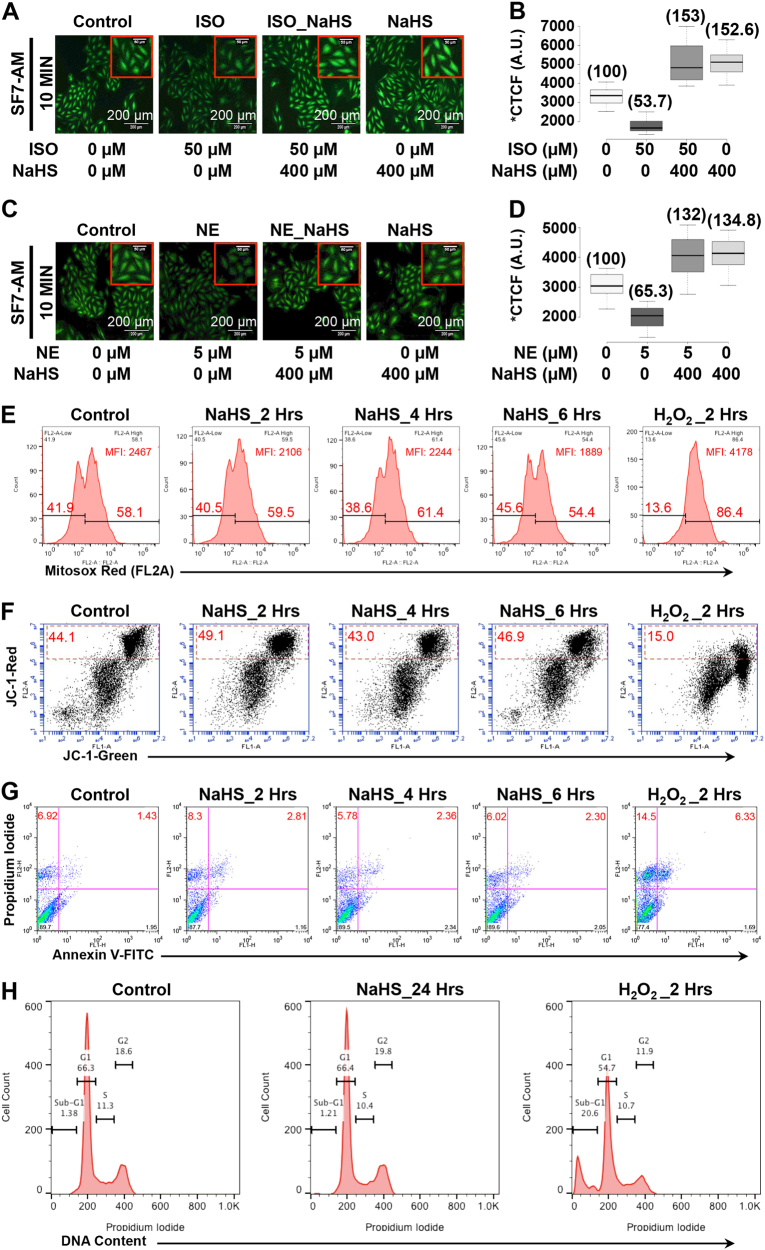
β-adrenergic stimulation downregulates endogenous H_2_S levels **a**–**d** The cells were stained with H_2_S-specific dye, SF7-AM (2.5 µM) and relative levels of endogenous H_2_S estimated in cardiac cells, with or without β-AR stimulation (employing ISO/NE), in the presence or absence of NaHS pre-treatment (400 µM, 30 min). The representative fluorescence micrographs (10X magnification, *λ*_ex/em_: 495/520 nm) for specific groups (*n* = 3) are shown in panels **a** and **c**. Magnified regions (inset) and scale bar is also shown for images in all groups. Panels **b** and **d** depict box-whisker plots representing corrected total cell fluorescence, CTCF, for indicated groups. The relative change in mean CTCF (represented as % of control) is also indicated in parenthesis. **e–h** The cells were treated with NaHS (400 µM) or H_2_O_2_ (50 µM) for indicated duration of time and specific assays were performed. **e** Mitochondrial superoxide measurement utilizing Mitosox red (mitochondrial superoxide-specific probe) staining and flow cytometry (BD Accuri C6) **f** Mitochondrial membrane potential (ΔΨm) assessment utilizing JC-1 dye and flow cytometry (BD Accuri C6). FL-1 (green) vs. FL-2 (red) dot plots were generated for the indicated groups. Cells with normal ΔΨm are detected as those with higher FL-2 signal (FL-1^bright^, FL-2^bright^ cells)—indicative of higher levels of JC-1 aggregates (detected in FL-2, 570 nm). The loss of mitochondrial membrane potential results in increased levels of FL-1^bright^, FL-2^dim^ cells—indicative of higher JC-1 monomer (detected in FL-1, 530 nm). **g** Apoptosis assay: the cells were subjected to Annexin V-FITC/Propidium Iodide (PI) double staining and data acquired using flow cytometry (BD FACS Calibur). FITC^−^/PI^−^ cells indicate normal cells while FITC^+^/PI^−^, FITC^+^/PI^+^ and PI^+^ suggest the presence of cells in specific stages of apoptosis (early, late, or necrotic). The percentage of cells in individual quadrant is also indicated in the figure. **h** DNA content analysis: the cells were stained with propidium iodide and DNA content analysis (cell cycle) done employing flow cytometry (BD FACS Calibur). Histogram plot for specific groups is as shown. The relative fractions of cells in specific stages of cell cycle (G1, S or G2) are also indicated. H_2_O_2_ treated group also showed distinctive sub-G1 population, indicating the presence of apoptotic cells.

β-adrenergic receptor (β-AR) overstimulation is known to culminate in left ventricular hypertrophy. In view of aforementioned inverse association between myocardial H_2_S levels and cardiac hypertrophy/dysfunction, we sought to investigate if β-AR stimulation per se can modulate endogenous H_2_S levels in cardiomyocytes. For the same, we stimulated H9c2 cells with isoproterenol (ISO, 50 μM) and measured the endogenous H_2_S levels employing a highly specific intracellular probe, SF7-AM. As shown in Fig. 1a, b, the stimulation of cells with ISO culminated in marked decrease in endogenous H_2_S levels, as early as 10 min post-stimulation. We additionally tested if the AR-agonist-induced decrease in the endogenous H_2_S levels could be prevented employing an exogenous donor. As shown in Fig. 1a, b, pre-treatment of the cells with 400 μM NaHS (30 min prior to adrenergic stimulation) reproducibly prevented β-AR agonist-induced decrease in the intracellular levels of H_2_S. Interestingly, we observed similar results when the cardiomyocytes were stimulated using another β-AR agonist, Norepinephrine (NE, 5 μM) (Fig. 1c, d) and thus, suggesting general biological relevance of this phenomenon downstream of β-AR stimulation.

Since pharmacological pre-treatment of the cells with NaHS elevated endogenous levels of H_2_S, significantly (by nearly 30–50%) over untreated cells (Fig. 1a–d); it was important to establish if this phenomenon produced any adverse effects, at early or extended durations of time, in these cells. For the same, we monitored chronic effects on cells, including changes in cell viability (employing Annexin V-PI staining) and growth pattern (as assessed by distribution of the cells in specific stages of cell cycle) besides acute cellular responses, including changes in mitochondrial ROS levels (employing mitochondrial-specific dye, MitoSOX Red) and mitochondrial membrane potential (utilizing JC-1). As a positive control (for determination of cytotoxic effects employing such assays), we utilized cells treated with 50 μM H_2_O_2_ (known to culminate in deleterious effects) and simultaneously subjected these to similar assays. As evident from time-dependent data presented in Fig. 1e–h, NaHS pre-treatment did not produce any detectable change in these parameters (mitochondrial ROS production, membrane potential, cell viability, or cell cycle pattern), neither at early nor late time points. In stark contrast, however, most of these parameters were significantly modulated in the cells treated with 50 μM H_2_O_2_, as early as 2 h post treatment. Taken together, these results unambiguously showed that the prophylactic, moderate elevation of endogenous H_2_S (employing NaHS) did not produce any deleterious/cytotoxic effects in these cells but was highly effective in preventing AR-agonist-induced decrease in endogenous H_2_S levels.

### Cellular H_2_S levels regulate adrenergic stimulation-induced hypertrophic responses

**Fig. 2:**
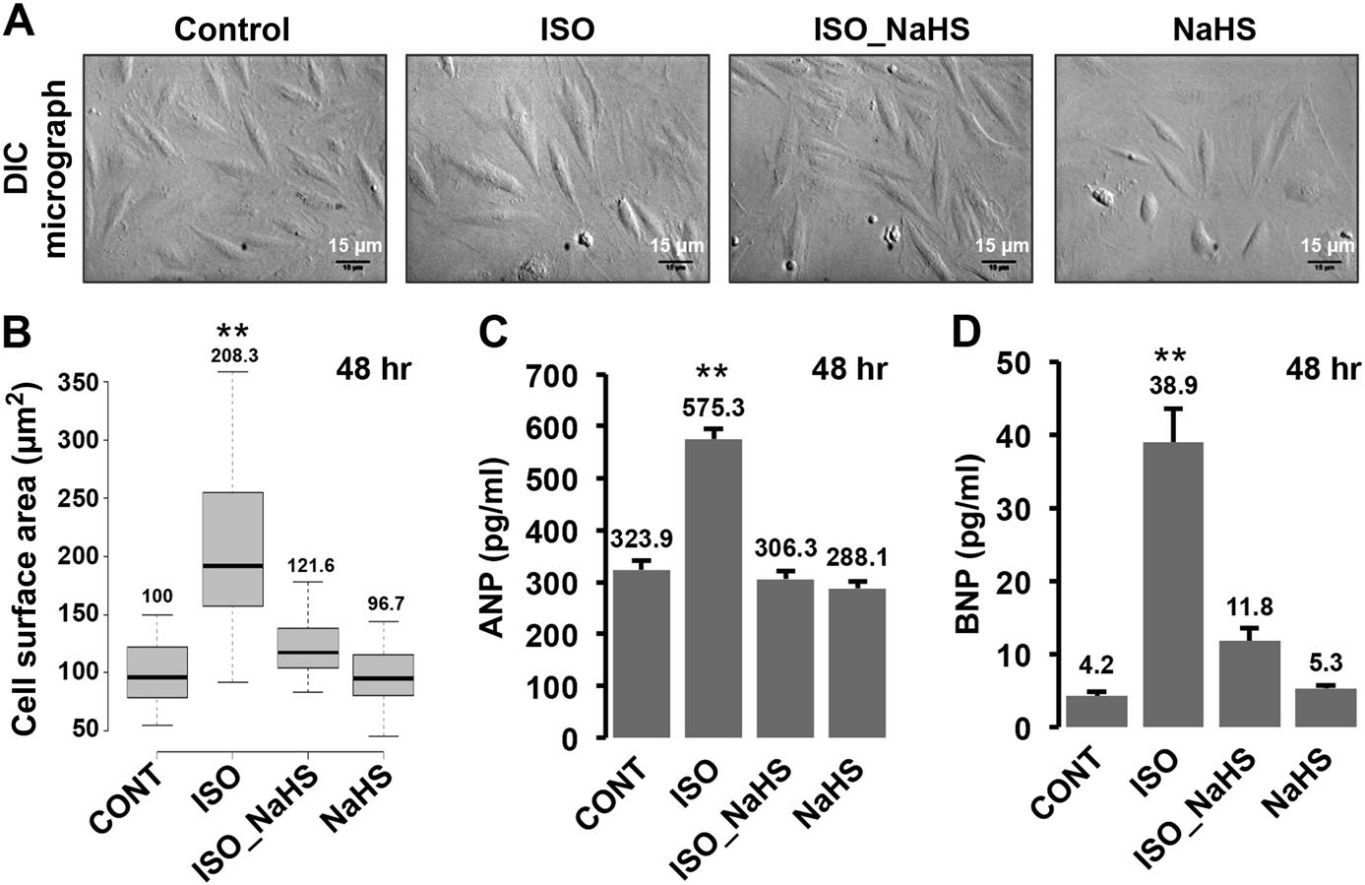
Augmentation of endogenous H_2_S suppresses β-adrenergic stimulation-induced hypertrophy in cardiomyocytes **a** DIC micrographs. H9c2 cells were treated with ISO for 48 h, with or without NaHS pre-treatment for 30 min and imaged utilizing DIC microscope (20X magnification). Scale bar is included in all images. **b** Box-whisker-plot showing cell surface area (in µm^2^) for specific groups, calculated using Image J, NIH. **c**, **d** The cells were challenged with ISO for 48 h, in the presence or absence of NaHS pre-treatment and culture supernatant collected for estimating secreted levels (in pg/ml) of ANP **c** and BNP **d**, utilizing sandwich ELISA. Mean ± S.E. was plotted to obtain the bar graphs shown in the figure. **p < 0.01

Since prophylactic elevation of endogenous H_2_S could prevent β-AR stimulation-induced decrease in the intracellular H_2_S levels, we next sought to establish potential functional implications of this phenomenon. For the same, we stimulated the cells with β-AR agonist, ISO, in presence or absence of NaHS pre-treatment (30 min prior to stimulation with ISO) and studied hypertrophic changes (employing differential interference contrast (DIC) microscopy) in these cells. As shown in Fig. 2a, b, we observed a significant increase (~200%) in the surface area of the cells (indicating hypertrophy), 48 h after ISO stimulation. Interestingly, pre-treatment of the cells with NaHS significantly prevented such hypertrophic response to ISO stimulation (Fig. 2a, b). These observations suggested critical, intrinsic role of β-AR stimulation-induced early decrease in endogenous cellular H_2_S levels during hypertrophic responses.

The cardiomyocytes characteristically respond by increasing the expression and secretion of atrial natriuretic peptide (ANP) and B-type natriuretic peptide (BNP) during hypertrophic stimulation^23,24^. We, therefore, additionally measured changes in the levels of secreted ANP and BNP in cell cultures, treated with isoproterenol, in presence or absence of NaHS pre-treatment. As shown in Fig. 2c, d, we observed a significant increase in the levels of ANP and BNP, 48 h after ISO treatment. This effect (of adrenergic stimulation) was, however, markedly reduced in the cells pre-treated with NaHS, prior to ISO stimulation. These results independently supported our proposition pertaining to critical role of endogenous H_2_S levels during adrenergic overstimulation-induced hypertrophy of cardiomyocytes.

### H_2_S modulates an integrated metabolic network regulating cellular redox homeostasis

**Fig. 3:**
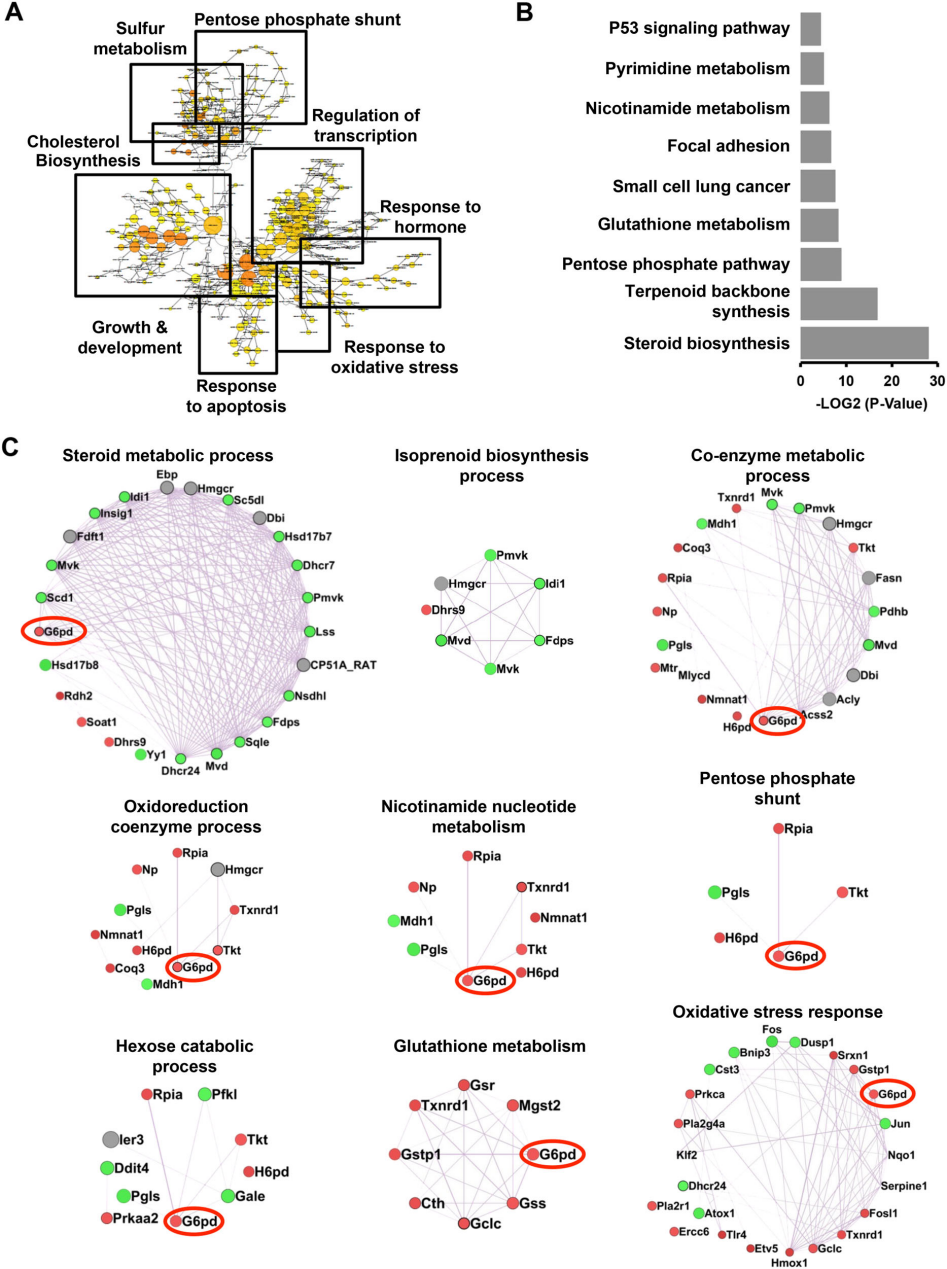
Genome-wide expression analysis suggests modulation of metabolic processes during H_2_S augmentation RNA sequencing analysis was performed on H9c2 cells, treated with NaHS for 6 h. The list of differentially expressed genes was subjected to analysis employing various softwares including BiNGO and GeneMANIA (as Cytoscape plugins) and DAVID (Online resource). The gene ontology (GO) networks were then represented as ‘‘Perfused forced directed clusters’’ and biological networks as ‘‘Degree sorted circular view.’’ **a** Clustered, over-represented GO and functional terms, utilizing hyper geometric test, in BiNGO (Cytoscape plug-in). The GO term clusters are indicated using closed boxes with gross specific annotation (indicated). **b** Bar graph representing −log_2_ (*p*-value) of significantly enriched KEGG pathways, as obtained from DAVID analysis. **c** Networks of co-expressed genes (representing significantly enriched biological processes) extracted utilizing GeneMANIA and shown as ‘‘Degree sorted circular view’’

To understand the molecular circuitry affected by H_2_S augmentation, we utilized an unbiased global transcriptome sequencing approach. We treated H9c2 cells with NaHS (400 μM) and generated differential transcriptome, 6 h post treatment. We observed 584 genes to be differentially expressed (≥1.4-fold, *p* < 0.05) in the cells treated with NaHS (Supplementary Table 1). Nearly 286 genes were upregulated and 298 genes downregulated, during these conditions. We, subsequently, subjected this data set to various data mining strategies*/*tools and identified specific biological processes and pathways—significantly over-represented in this data set. Interestingly, we observed common biological processes emerging in independent data mining strategies, suggesting specificity of analysis and also, unique nature of cellular responses to modulation of H_2_S levels. As shown in Fig. 3a, the clustering of biological terms (associated with individual differentially expressed genes), utilizing ‘‘BiNGO’’, revealed enrichment of basic biological processes such as pentose phosphate shunt, regulation of transcription, redox homeostasis, sulfur metabolism, and cholesterol biosynthesis amongst others. The list of significantly enriched ‘‘KEGG Pathways’’ (Fig. 3b, Supplementary Fig. 1A–J, Supplementary Table 2) grossly recapitulated a similar biological theme—with pathways including steroid biosynthesis, terpenoid backbone synthesis, pentose phosphate pathway (PPP), nicotinamide nucleotide metabolism, glutathione metabolism and p53 signaling pathway significantly enriched in this data set.

We next extracted network of co-expressed genes regulating such pathways, utilizing ‘‘Gene MANIA.’’ This analysis (Fig. 3c), besides returning an identical biological theme, revealed additional striking aspects: (1) Majority of the networks were associated with metabolic or redox homeostasis processes. (2) Within such metabolic processes, three discrete pathways were readily discernable. These included steroid/isoprenoid biosynthesis pathway, oxidoreductase coenzyme metabolic pathway (principally representing pentose phosphate pathway) and glutathione metabolism pathway (a previously known target of H_2_S). (3) Taken together, H_2_S appeared to modulate an integrated cellular metabolic circuitry—centrally linked by nicotinamide nucleotide cofactor (NADPH) homeostasis. This integrative view appears pertinent in consideration of the fact that while PPP is the key cellular pathway generating NADPH (reduced form), cholesterol biosynthesis and glutathione metabolism are the major cellular pathways consuming cellular reducing power (NADPH). (4) G6PD—the first and rate-limiting enzyme within PPP—stood as the highest degree node (highlighted in Fig. 3c) within these sub-networks and thus, suggesting it to be of key significance (node) for biological effects produced by H_2_S.

### Functional evidences for modulation of G6PD activity by endogenous H_2_S augmentation

**Fig. 4:**
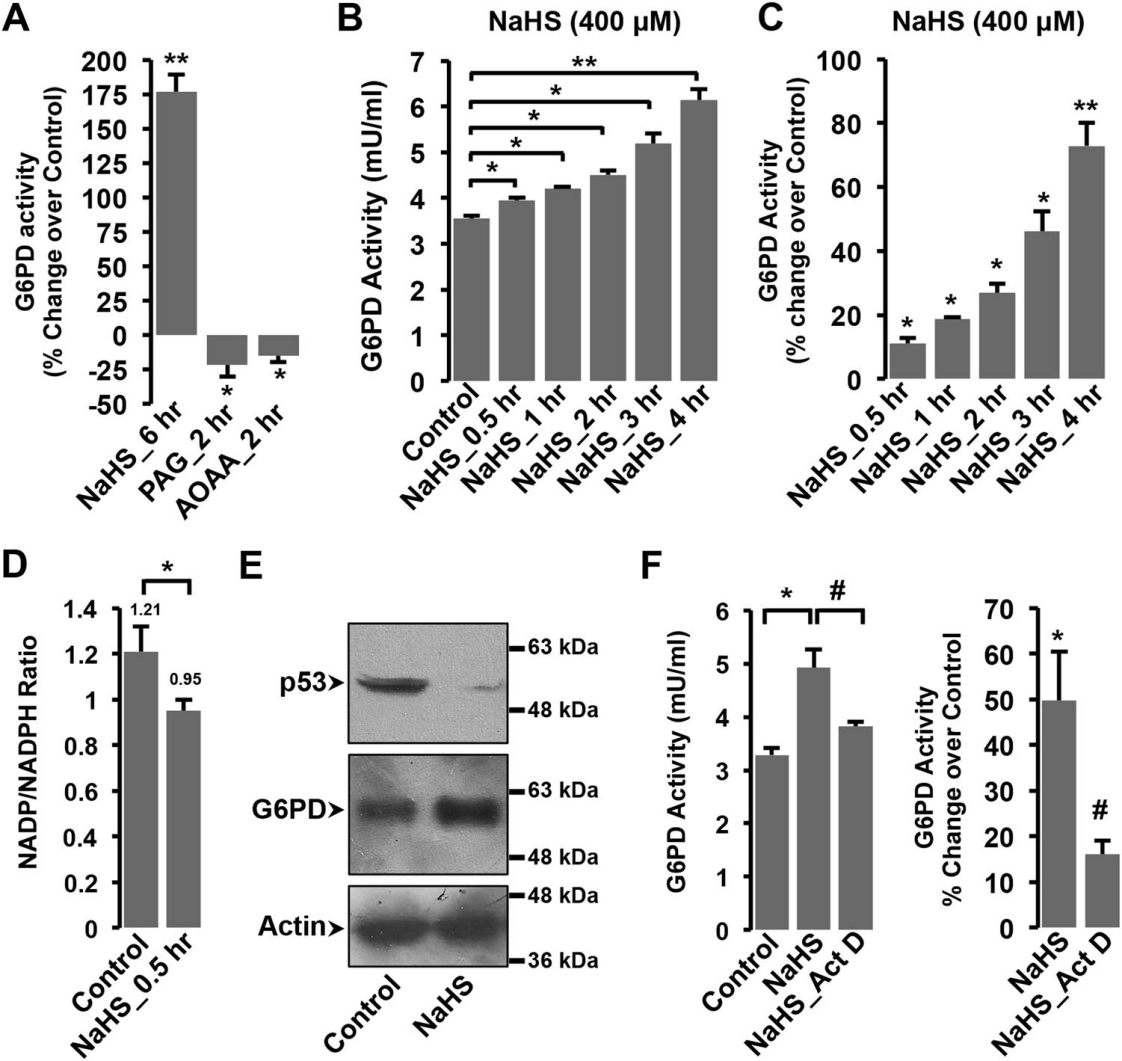
Functional evidences for regulation of G6PD by intracellular H_2_S levels **a** Bar graph representing G6PD activity (as percentage change over control) in cells treated with NaHS (400 μM, 6 h), PAG (1 mM, 2 h), and AOAA (2 mM, 2 h). **b**, **c** Bar graphs representing time-dependent changes in G6PD activity (mU/ml) **b** and percentage change over control **c**, post 400 μM NaHS treatment for indicated duration of time. One unit of G6PD activity was defined as the amount of enzyme that catalyzed the conversion of 1.0 µmol of glucose-6-phosphate into 6-phosphoglucono-δ-lactone and converted 1.0 µmol of NAD^+^ to NADH per minute at 37 °C. **d** Bar graph representing NADP/NADPH ratio in H9c2 cells, 0.5 h post NaHS treatment. **e** Representative immunoblots for p53 or G6PD expression in whole-cell extracts from cells treated with or without NaHS. β-actin was used as loading control. **f** G6PD activity (represented in mU/ml or percentage change over control) for cells treated with NaHS (400 μM, 4 h), in the presence or absence of actinomycin D (5 µg/ml, 2 h prior to NaHS treatment). Mean ± S.E. was plotted to obtain bar graph shown in the figure. *p < 0.05, **p < 0.01. ^#^Compared to NaHS groups

We, next, sought to generate functional evidence for modulation of G6PD activity by endogenous H_2_S levels. For the same, besides NaHS treatment (to elevate endogenous H_2_S levels), we additionally treated the cells with PAG & AOAA (inhibitor of CSE & CBS—two key enzymes responsible for endogenous generation of H_2_S) to decrease cellular H_2_S levels. As shown in Fig. 4a, we observed significant increase in G6PD activity with the elevation of endogenous H_2_S levels (NaHS pre-treatment, 6 h). Interestingly, the treatment of cells with PAG and AOAA alone significantly reduced G6PD activity, below that observed in untreated cells (Fig. 4a). Taken together, these observations suggested an intrinsic, direct relationship between endogenous H_2_S levels and cellular G6PD activity.

We, additionally, monitored G6PD activity in a time-dependent manner, post NaHS stimulation. Intriguingly, we observed significant increase in G6PD activity, as early as 30 min post NaHS treatment and increasing gradually with time (Fig. 4b, c). Since G6PD is critical for generating NADPH, we next sought to generate functional proof for elevation of G6PD activity, at the earliest time point (30 min), by estimating cellular NADP/NADPH ratio. As shown in Fig. 4d, we observed a significant increase in NADPH levels (and thus, lower NADP to NADPH ratio) in the cells treated with NaHS. This observation yielded functional evidence for rapid modulation of G6PD activity in response to H_2_S augmentation.

Our transcriptome data also suggested modulation of p53-regulated pathway (Fig. 3b and Supplementary Table 2), after NaHS treatment. Notably, p53 protein is known to interact with G6PD and regulate its activity by preventing formation of active dimers^25^. We, therefore, additionally monitored cellular levels of p53 in the cells treated with NaHS. As shown in Fig. 4e, we observed significant reduction in the cellular level of p53 protein, 4 h post NaHS treatment. Interestingly, the expression of G6PD protein was elevated at this time point (Fig. 4e). In view of our transcriptome data (suggesting upregulation of G6PD transcript, Fig. 3, Supplementary Table 1), we further, treated the cells with actinomycin D and measured G6PD activity, 4 h post NaHS treatment. The NaHS-induced increase in G6PD activity could be significantly prevented with actinomycin D treatment (Fig. 4f), supporting the possibility of de novo synthesis of G6PD, post NaHS treatment. It thus appears reasonable to suggest that NaHS-induced sustained elevation of G6PD activity over extended duration of time (as evident from Fig. 4) could be due to potentiation of multiple processes/pathways (by NaHS), including increase in active dimers (due to lowered levels of inhibitory protein, p53) and its de novo (elevated) expression (Figs. 3 and 4e, f).

### Critical role of elevated cellular G6PD activity during suppression of β-AR-induced hypertrophic effects by H_2_S

**Fig. 5:**
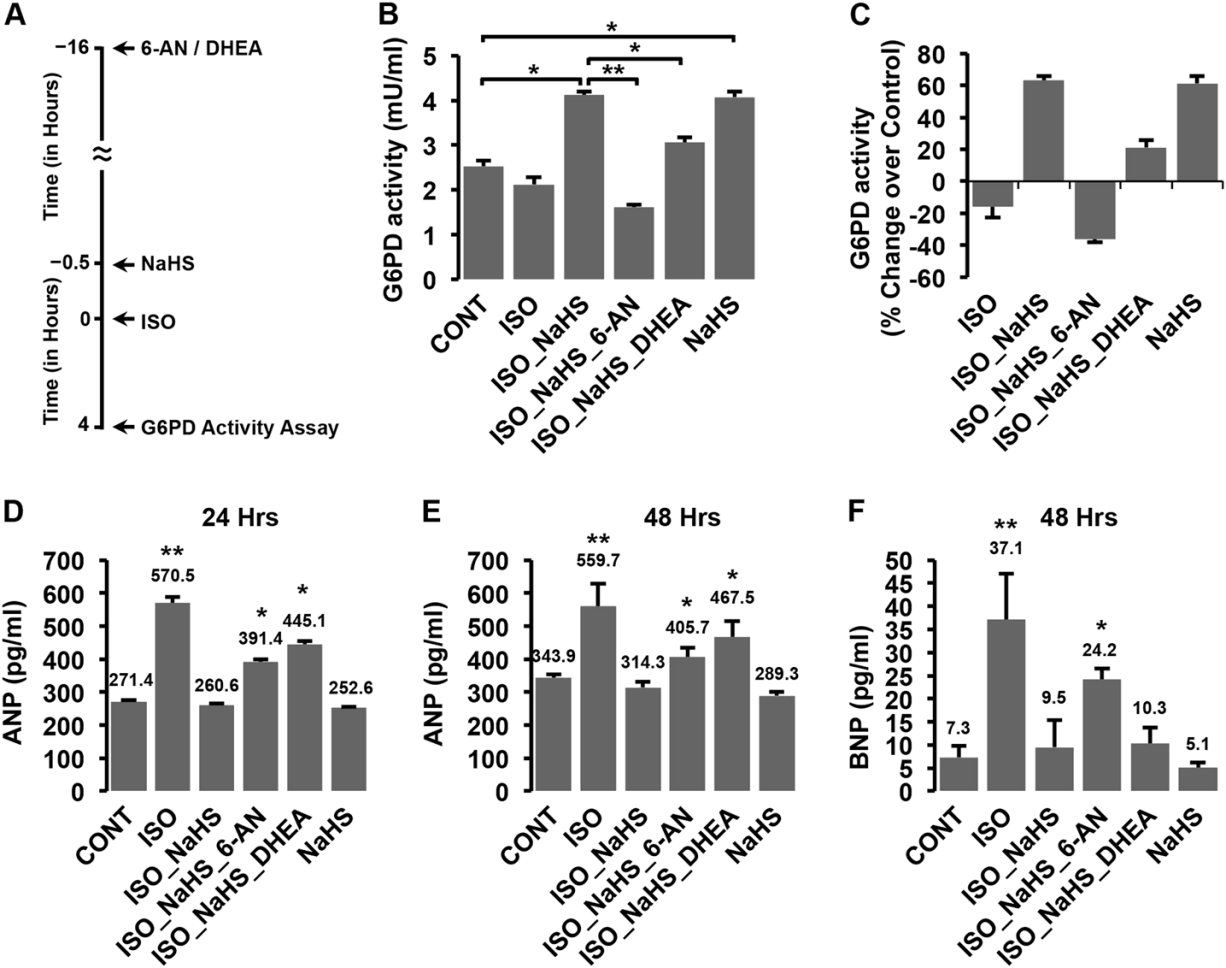
H_2_S-induced increase in G6PD activity is critical for regulating hypertrophic responses **a** Schematic representation for experimental set in panel **b** and **c**. H9c2 cells (with or without 30 min NaHS pre-treatment) were stimulated with ISO (50 µM), in the presence or absence of inhibitors; 6-AN (250 µM, 16 h) or DHEA (25 µM, 16 h). **b**, **c** Bar graphs (Mean ± S.E.) representing G6PD activity in mU/ml **b** or percentage change over control **c** from various groups (as indicated in figure). **d**–**f** Bar graph (Mean ± S.E.) depicting ANP or BNP levels (in pg/ml), as estimated from culture supernatants after 24 **d** or 48 h **e**, **f** of ISO treatment, with or without NaHS, in the presence or absence of G6PD inhibitors, 6-AN or DHEA. *p < 0.05, **p < 0.01 compared to control groups

We, next, sought to investigate if H_2_S-induced increase in the activity of G6PD was causally involved in inhibiting β-AR-induced hypertrophy. For the same, we utilized empirically determined doses of two-specific pharmacological inhibitors of G6PD, namely 6-AN (a competitive inhibitor) and DHEA (a non-competitive inhibitor), in the cells pre-treated with NaHS and challenged those with ISO (Fig. 5a). As evident from Fig. 5b, c, NaHS significantly increased G6PD activity in cells with or without ISO treatment. Interestingly, the pre-treatment of cells with either 6-AN or DHEA curtailed such NaHS-induced increase in G6PD activity and kept it significantly below those observed in NaHS (without G6PD inhibitor) treated groups (Fig. 5b, c). Under similar conditions, we next measured the levels of secreted ANP and BNP (as a measure of hypertrophic responses) after ISO stimulation (24 or 48 h) of these cells. As evident from Fig. 5d–f, while NaHS treatment (without G6PD inhibitors) suppressed ISO-induced elevation of ANP and BNP, significantly higher levels of these proteins (ANP and BNP) were observed in ISO-challenged groups with G6PD inhibitors despite NaHS pre-treatment. These observations suggested that G6PD was critical for manifestation of anti-hypertrophic effects of NaHS.

### Augmentation of endogenous H_2_S modulates G6PD activity and p53 levels in rat model system

**Fig. 6:**
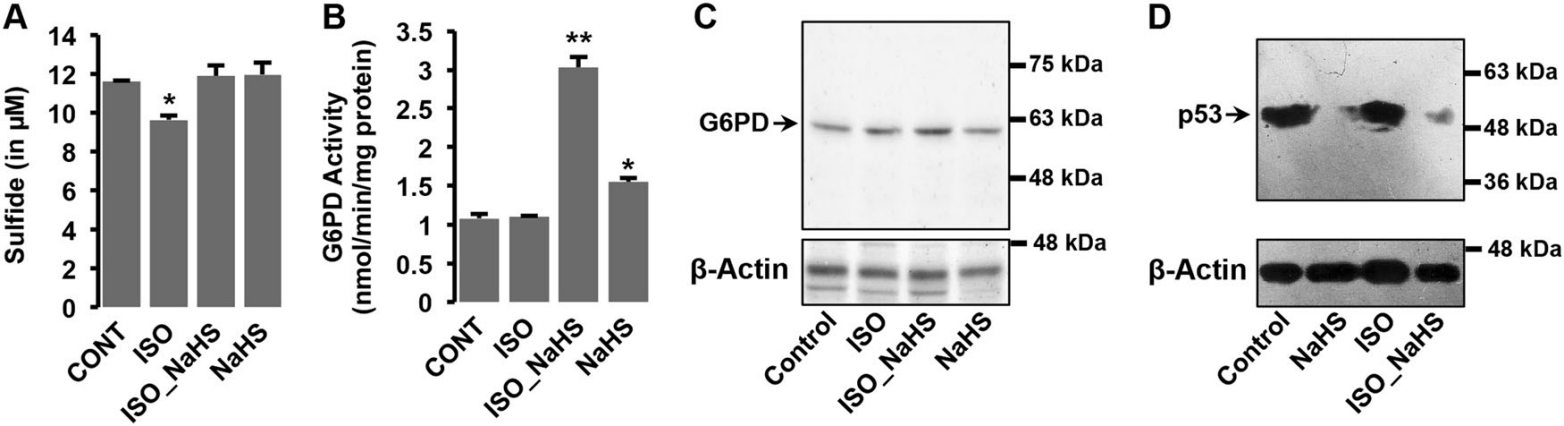
H_2_S augmentation modulates G6PD activity and expression in rat heart tissues Sham- or NaHS-treated animals were challenged with isoproterenol (5 mg/kg, s.c, 4 h) and specific assays performed. **a** Bar graph showing total sulfide concentration in heart tissues isolated from the indicated groups. **b** Bar graphs representing G6PD activity (in nanomoles (nmol) of NADPH (reduced form) produced per minute per mg of total protein) **c**, **d** Representative immunoblot for G6PD expression **c** and p53 expresssion **d** in heart tissues from indicated groups. β-Actin was used as the internal loading control. Mean ± S.E. values were used to plot the bar graph. *p < 0.05, **p < 0.01 compared to control groups

We next sought to investigate if H_2_S supplementation modulated similar pathways in animals during normal or stress conditions. We utilized an established rat model system of β-AR-induced cardiac hypertrophy^26^ and challenged the animals with low dose (5 mg/kg body weight per day) of isoproterenol (ISO), with or without NaHS pre-treatment (4 mg/kg body weight per day for 12 days, see methods for details). As shown in Fig. 6a, administration of isoproterenol (4 h) to the animals reduced endogenous levels of H_2_S in myocardial tissues. Notably, further, similar to the cultured cells, pre-treatment of the animals with NaHS effectively prevented ISO-induced decrease in cardiac H_2_S levels (Fig. 6a). Interestingly, the cardiac G6PD activity (Fig. 6b) and expression (Fig. 6c) was markedly elevated in the animals pre-treated with NaHS (12 days), with or without ISO challenge (4 h). We also studied the effects of NaHS pre-treatment (H_2_S augmentation) on cardiac p53 levels. As shown in Fig. 6d, the levels of p53 were also markedly reduced with NaHS supplementation, with or without ISO administration. These results, taken together, suggested that the effects of H_2_S augmentation were conserved under physiologically relevant conditions and manifested comparably (to those observed in cultured cells) in our animal model as well.

### H_2_S supplementation suppresses β-AR-induced cardiac hypertrophy, in G6PD-dependent manner, in rat model system

We next sought to investigate if NaHS could modulate cardiac hypertrophy induced by β-AR stimulation and if G6PD was causally involved in such effects in our animal model. For the same, we utilized 6-AN (specific G6PD inhibitor) in the animal model and studied the effect of H_2_S augmentation in conferring protection against β-AR stimulation (ISO) induced cardiac hypertrophy, with or without this inhibitor. Since this inhibitor can produce adverse effects (especially related to central nervous system and motor weakness) in cumulative dosage-dependent manner^27^, we first empirically established a non-toxic dose of 6-AN. We observed that four uniformly spaced doses of 1 mg/kg body weight (during entire experimental span), after every 5 days, did not produce any detectable adverse effects per se—neither grossly (activity/motility, survival, body weight, feed) nor in the heart tissues (morphological and histological parameters, Supplementary Fig. 2).

**Fig. 7:**
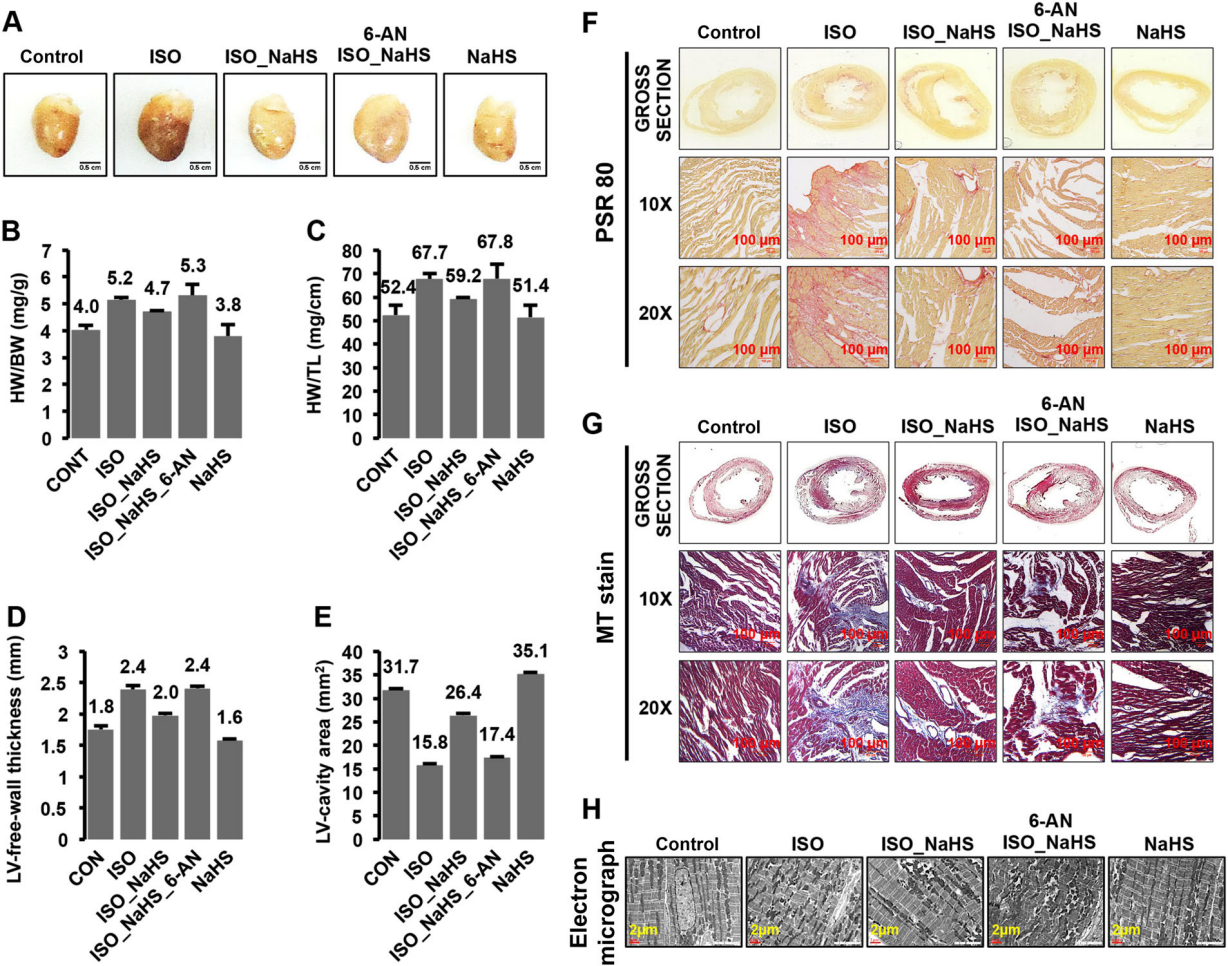
Critical role of H_2_S-induced increase in G6PD activity for suppression of β-AR-mediated cardiac hypertrophy in rat model The animals (NaHS- or Sham-treated), with or without 6-AN, were challenged with isoproterenol for 6 days and specific parameters studied. **a** Gross heart images, from indicated groups, representing the specific morphological changes. **b**, **c** Bar graphs (Mean ± S.E.) depicting heart weight (HW):body weight (BW), in mg/g, and heart weight (HW):tail length (TL), in mg/cm, in the indicated groups. **d**, **e** Bar graphs (Mean ± S.E.) representing left ventricular-free wall thickness (in mm) and left ventricular cavity area (in mm^2^), as estimated from sections of heart isolated from indicated groups. **f**, **g** Bright-field micrographs of Picro-Sirius Red (PSR 80, panel **f**) staining (Red—Collagen, Yellow—Muscle fibers/Cytoplasm), and Masson Trichrome (MT, panel **g** staining (Blue—Collagen, Red—Muscle fibers/Cytoplasm). **h** Transmission electron micrographs (Scale bar: 2 µm) of sections from left ventricular-free wall of the indicated groups

The circulating catecholamines mediate pressure-overload-induced left ventricular hypertrophy^28^. Similarly, in vivo stimulation of β-ARs employing exogenously administered catecholamines (ISO or NE) typically culminates into pressure-overload hypertrophy^26,29^—characterized by concentric increase in cardiac mass, manifesting as increase in the ventricular-free wall thickness, decrease in ventricular cavity cross-sectional area, lowered endo-cardial capillary density concomitant with marked ventricular fibrosis in left heart^30^. We carefully studied such parameters in specific groups, as described above, and represented these in Fig. 7a–h. Figure 7a depicts representative images of gross heart isolated from various groups. As shown in Fig. 7b, c, the cardiac mass (evident from heart-to body weight and heart weight-to tail length ratios) was increased in animals treated with isoproterenol and significantly lowered in those pre-treated with NaHS. The effect of NaHS pre-treatment on isoproterenol-induced increase in cardiac mass was attenuated with the administration of 6-AN (Fig. 7b, c), suggesting key role of G6PD in this process.

Figure 7d, e represent the average left ventricular-free wall thickness and LV cavity area (as estimated from cross-sections of heart tissues) from individual groups. In agreement with the characteristics of pressure-overload hypertrophy, we observed increase in left ventricular wall thickness and decrease in LV cavity area with the administration of isoproterenol to experimental animals (6 days). Strikingly, however, this phenomenon was significantly ameliorated in the animals pre-treated with NaHS. Interestingly, similar to the changes observed for cardiac mass, administration of 6-AN significantly inhibited protection conferred by NaHS under these conditions (Fig. 7d, e).

We, next, performed two different staining, namely PSR80 and MT, to estimate cardiac fibrosis in various groups of animals. As shown in Fig. 7f, isoproterenol treatment significantly increased collagen content in the heart (evident as red colored regions in PSR80-stained heart sections). The pre-treatment with NaHS significantly reduced collagen deposition in response to isoproterenol challenge but this effect was curtailed when 6-AN was additionally administered to the animals (Fig. 7f). Further, as evident from Fig. 7g, similar results were also obtained in MT staining of the heart tissue sections from various groups of animals (note that the collagen rich areas are stained blue in MT stained sections). We thus inferred that H_2_S-induced increase in G6PD activity was critical for its cardio-protective effects in the animal model of hypertrophy.

Next, we studied sarcomeric structural organization (critical for cardiac function) at ultra-structural level, employing electron microscopy, from various groups of animals described above. As shown in Fig. 7h, we observed sarcomeric disarray—the loss of typical sarcomeric organization (distinct acto-myosin banding pattern, evident as light and dark bands, and mitochondria, evident as electron dense structures in EM, arranged parallel to each unit)—in the cardiac tissues of animals administered isoproterenol for 6 days. Strikingly, pre-treatment of the animals with NaHS prevented this phenomenon (sarcomeric disarray with isoproterenol treatment). The inhibition of G6PD (employing 6-AN) counteracted such effect of NaHS—in preventing sarcomeric disarray resulting from isoproterenol treatment of the animals (Fig. 7h).

Our results, taken together, thus yielded strong evidence for regulation of a specific cardiac metabolic circuitry (involving G6PD as a key node) by H_2_S and its functional role in suppression of pathological β-AR stimulation—progressively culminating into cardiac hypertrophy.

**Fig. 8:**
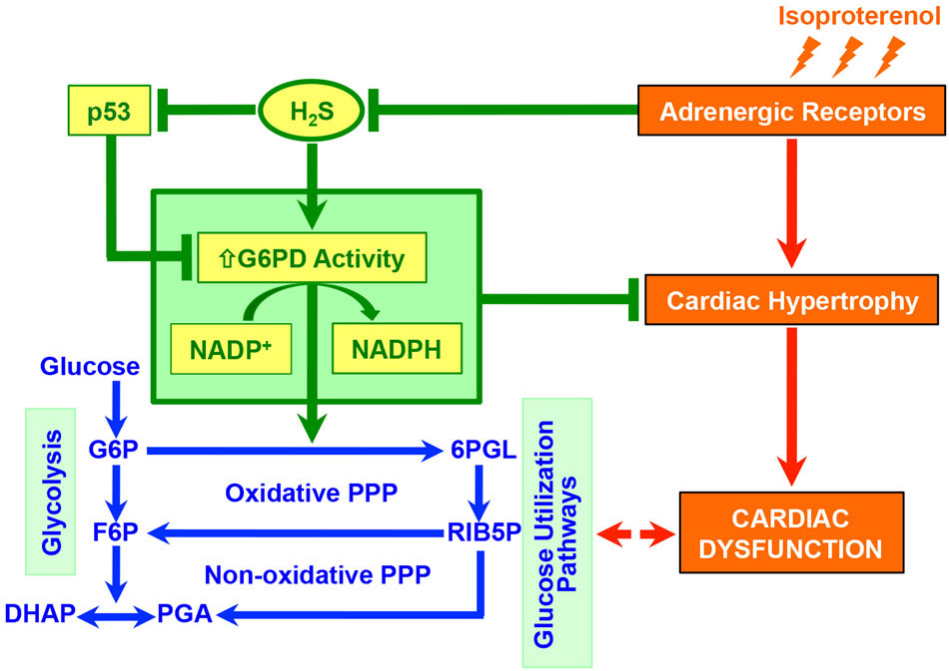
Diagrammatic representation of the phenomenon deciphered from the work, conceptually integrated to known framework of cardiac hypertrophy and basic glucose metabolic pathway β-adrenergic receptor (β-AR) stimulation—known to culminate in cardiac hypertrophy—rapidly lowers endogenous levels of H_2_S in cardiomyocytes. H_2_S is intrinsically linked (directly) to G6PD activity (and production of NADPH), via multiple processes, and this phenomenon is causally involved in suppression of cardiac hypertrophy (by H_2_S) in both cellular and animal model systems. Progression of cardiac hypertrophy and ensuing dysfunction is, per se, known to be associated with modulation of glucose utilization pathway. G6PD: glucose-6-phosphate dehydrogenase, G6P: glucose-6-phosphate, 6PGL: 6-phospho-gluconolactone, F6P: fructose-6-phosphate, RIB5P: ribulose-5-phosphate, DHAP: dihydroxy acetone-phosphate, PGA: glyceraldehyde-3-phosphate

Figure 8 depicts an integrative view of phenomenon deciphered from our present work (see legend for details), in close association with the known, major glucose metabolic pathway.

## Discussion

We here presented evidence that pathological adrenergic responses in cardiac cells ensue by downregulating endogenous levels of H_2_S. An exogenous donor—which annulled such effect by maintaining stable levels of intracellular H_2_S under these conditions—markedly attenuated pathophysiological effects of adrenergic overstimulation, in both cellular as well as animal model systems. Our data further suggested that endogenous H_2_S is physiologically coupled to an integrated cellular metabolic network, centrally connected by nicotinamide dinucleotide cofactors, in cardiomyocytes. H_2_S modulated production of NADPH by regulating G6PD activity through at least two putative processes—elevated G6PD expression (transcript as well as protein) and downregulation of p53 protein (which negatively regulates G6PD activity by preventing formation of active enzyme dimers). The inhibition of G6PD significantly curtailed the effects of H_2_S in suppressing β-AR-induced hypertrophy, in both cellular and animal model. These observations hence demonstrate G6PD as a critical mediator of H_2_S-induced cardioprotective effects.

The myocardial tissue, interestingly, is uniquely posed with regards to G6PD and PPP. Despite high metabolic activity, the basal G6PD activity and PPP output in rat heart has been shown to be significantly lower than those observed in other vital organs systems, kidneys, and liver^31,32^. Somewhat comparable cardiac G6PD activity was also reported for humans^32,33^, plausibly implicating an evolutionarily conserved phenomenon in heart. The cardiac cells, further, appear to maintain high levels of isocitrate dehydrogenase—an alternate enzyme (mitochondrial) capable of generating NADPH^34^. Notably, however, there exists multiple lines of compelling evidence to surmise a critical role of myocardial G6PD and PPP. First, the inhibition of G6PD impairs contractile function in cultured cardiomyocytes^35^, likely through inhibition of l-type calcium channels^36^. G6PD-deficient (mutant) animals, interestingly, exhibit early cardiac remodeling and dysfunction compared to normal littermates^35^. Such animals are also prone to accelerated cardiac hypertrophy in response to pressure overload and infarct^37^. Second, there is an intrinsic propensity for increase in G6PD activity, though a significantly delayed response (after 12 h), to sustained adrenergic stimulation^31^. Third, the augmentation of G6PD activity—either through pharmacological intervention such as dichloroacetate, DCA,^38,39^ or genetic means to increase G6PD activity or PPP flux^40^—bestows an explicit protective advantage against cardiac hypertrophy. In view of such information and the observations described in our present work, it is reasonable to conclude that the unfailing cardio-protective efficacy of H_2_S has a robust mechanistic basis—the intrinsic regulation of expression/activity of an enzyme (G6PD) occupying evolutionarily conserved, critical niche in myocardial function and bioenergetics.

Besides NADPH generation, another important function of PPP includes the production of ribose 5-phosphate, critical for synthesis of de novo purine nucleotides^41^. Ribose 5-phosphate, in turn, feeds the cellular pool of 5-phosphoribosyl-1-pyrophosphate (PRPP). During increased energy demand, such as that during adrenergic stimulation, the cellular PRPP levels are extremely important (and can become limiting) for maintaining cellular ATP pool^32,42^. Not surprisingly, therefore, ribose supplementation has been shown to produce beneficial effects during various pathological conditions^42–44^. In view of such information, it is likely that the increased PPP flux, in response to H_2_S augmentation, could elevate ribose levels and thus, cumulatively contributing to suppression of cardiac hypertrophy in response to catecholamine stimulation.

The onset and progression of cardiac hypertrophy, per se, is characteristically marked by cellular metabolic reprogramming—involving downregulation of β-oxidation pathways and increased reliance on glucose utilization^45,46^. Accumulating literature suggests that such fetal metabolic signature (dependence on glucose for ATP generation) is essentially pro-adaptive and its inhibition further aggravates cardio-pathological outcomes^40^. Several of such metabolic genes are known targets of HIF-1α—a key transcription factor regulating glycolytic pathway^47^. Notably, further, HIF-1α also regulates (in a p53-dependent manner) critical genes involved in cardiac angiogenesis^48^—another critical phenomenon regulating cardiac failure^49^ and dependent on H_2_S^50^. Strikingly, the inhibition of HIF-1α causes cardiac dysfunction during pressure overload^51^. The stabilization and biological activity of HIF-1α is, thus, of unambiguous significance in governing pathological/deleterious outcomes associated with hypertrophic cues. Interestingly, a recent report suggested that pentose phosphate pathway and cellular NADPH levels are critical for stabilization of HIF-1α and expression of its target genes^51^. Taken together, it is thus reasonable to suggest that H_2_S-induced augmentation of G6PD (and elevated cellular NADPH levels) would positively support stabilization of HIF-1α—a multifaceted adaptive cardiac response—during sustained adrenergic stimulation and cardiac remodeling.

It is also important to note that cellular NADPH can also be utilized, paradoxically, by cardiac Nox isoforms^52–54^ (cytoplasmic/mitochondria) for generation of ROS—known to be involved in pathological adrenergic signaling and other deleterious effects^55–57^. In our experiments, however, we did not observe increase in intracellular ROS in cells treated with NaHS (Fig. 1e), despite marked increase in NADPH under these conditions (Fig. 4d). A likely explanation for such observation could be the fact that besides generation of reducing equivalents, H_2_S augmentation culminated in ‘‘active routing’’ of cellular reducing power towards GSH metabolic pathway—through elevated expression of multiple genes, including *Gclc, Gss, Mgst 2, Gsr, Gstp 1, Cth, and Txnrd1* (Fig. 3, Supplementary Fig. 1A-J & Supplementary Table 1 & 2). Interestingly, further, the transcript of an important cardiac-specific, ROS-producing enzyme, *Nox 4*, appears to be downregulated in the cells stimulated with β-AR agonists in the presence of NaHS pre-treatment (Chhabra et al., unpublished data) and thus, likely limiting endogenous pathways utilizing NADPH for ROS generation per se. In view of such observations, cumulatively, we hypothesize that modulation of endogenous H_2_S uniquely orchestrates synergistic pathways—comprising of ‘‘NADPH-production’’ and ‘‘downstream-utilization’’ processes—and re-defines the cellular ‘‘redox threshold’’ critical for pathological adrenergic signaling in cardiomyocytes.

In conclusion, our results, besides describing critical mechanistic basis, implicate an important intrinsic role for H_2_S in regulating cellular ‘‘metabolic state’’ and thus, soliciting further studies to explore if endogenous modulation of this gaseous messenger could be at the ‘‘heart’’ of other diseases, intrinsically involving metabolic perturbations.

## Materials and methods

### Cell culture and pharmacological treatments

Embryonic rat heart-derived cardiomyoblast cell line, H9c2, was purchased from Sigma Aldrich and cultured in high glucose (4500 mg/l) containing Dulbecco’s modified Eagle’s Medium (DMEM, D7777, Sigma Aldrich) supplemented with 10% (*v/v*) fetal bovine serum (FBS), 100 U/ml penicillin and 100 μg/ml streptomycin. Cells were maintained at 37 °C in a humidified incubator with 5% CO_2_. The culture medium was replaced with fresh media every 48 h and the cells were sub-cultured, when the cultures approached 70–80% confluency. Cells were shifted to serum- and antibiotic-free media (DMEM without FBS and Penicillin-Streptomycin) 16 h prior to agonist/drug treatment. For H_2_S treatment, H9c2 cells were pre-treated with 400 µM Sodium hydrogen sulfide (NaHS, Cayman Chemicals), prepared fresh in PBS, for 30 min prior to adrenergic stimulation. The adrenergic stimulation was done employing freshly prepared 50 µM Isoproterenol (ISO, Sigma) in normal saline, for the indicated duration of time. For CSE and CBS inhibition, the cells were treated with 1 mM L-C-Propargylglycine (PAG, Sigma) or 2 mM Aminooxyacetic acid hemihydrochloride (AOAA, Sigma), respectively. For actinomycin D treatment, cells were pre-treated with 5 µg/ml of drug, prepared in DMSO. The cells were treated with 25 µM Dehydroepiandrosterone (DHEA, Sigma) or 250 µM 6-Aminonicotinamide (6-AN, Sigma), for G6PD inhibition.

### Microscopy and quantitation

Fluorescent imaging was carried as per standard protocol and guidelines, briefly described below. The images were acquired using Motic AE31 microscope (attached with camera, Moticam 2500, China). The acquisition settings, including parameters such as exposure time, gain, gamma correction amongst others, were strictly kept constant for acquisition of images from specific groups within an individual experiment. A minimum of six independent fields was acquired, at 10X magnification, for each specific group. For quantification of images, unprocessed raw microscopic images were used and the cell fluorescence measured utilizing Image J, NIH. The corrected total cell fluorescence (CTCF) was calculated as Integrated Density—(Area of selected cell × Mean fluorescence of background readings) for a minimum of 25 cells per group, from at least six independent fields^58^. The CTCF values were then plotted as box- and whisker-plot using BoxPlotR^59^. The DIC images were acquired utilizing inverted DIC microscope (Axiovert-200, Carl Zeiss). Cell surface area (in μm^2^) was estimated using Image J (NIH). The statistical significance between observations from specific groups within an experiment was established by one-way analysis of variance. A minimum of three independent biological repeats was performed for all individual experiments.

### Intracellular H_2_S estimation (SF7-AM staining)

Cells were pre-loaded with 2.5 µM Sulfidefluor 7-AM (SF7-AM), Tocris Bioscience and incubated at 37 °C in CO_2_ incubator for 30 min. NaHS (400 µM) treatment was given to the designated groups for 30 min. Subsequently, the cells were stimulated (in serum- and phenol red-free media) for 10 min with 50 µM Isoproterenol (ISO), Sigma or 5 µM Norepinephrine (NE), Sigma, as required in individual experiments. Images were acquired at 10X magnification (as described above) using Motic AE31 microscope (*λ*_ex/em_: 495/520 nm).

### Mitochondrial superoxide and membrane potential estimations

To estimate mitochondrial superoxide anion, the cells were stained with 5 µM MitoSOX Red (Molecular Probes) for 15 min. For mitochondrial membrane potential (ΔΨm), JC-1 dye (T3168, Thermo Fisher Scientific) was employed at a concentration of 5 µg/ml. The cells were subsequently acquired using BD Accuri C6 cytometer equipped with 488 nm excitation and 530/585 nm band pass emission filter.

### Cell viability and cell cycle analysis

Cell viability assay was performed utilizing Annexin V/Propidium Iodide staining kit (Sigma) and the cell cycle analysis was done as per our previously published protocol^60^, utilizing FACS Calibur (BD).

### Enzyme-linked immunosorbent assay (ELISA)

The levels of ANP and BNP in cell culture media were measured by sandwich ELISA, employing commercially available kit (Rat ANP ELISA kit, ab108797; Rat BNP 45 ELISA kit, ab108816, Abcam) as per the manufacturer’s protocol.

### RNA isolation, sequencing, and bioinformatic analysis

Total RNA was extracted from the cells utilizing Trizol reagent (Sigma Aldrich) and purified employing RNeasy Mini Kit (Qiagen, Hilden, Germany) with on-column DNase digestion. RNA sequencing, annotation, differential gene expression and data mining (Bioinformatic analysis) was done as per protocols described by us previously^61,62^.

### G6PD activity assay

The homogenates from cell (4% w/v) or heart tissue (8% w/v) were prepared in ice cold PBS by sonication (30% amplitude, ten cycles of 5 s sonication with 5 s gap) using Ultra-Sonicator (VC-505, Sonics Vibra Cell, USA). Samples were centrifuged at 13,000 rpm for 10 min and supernatant collected. The assays were performed utilizing G6PD activity kit (ab102529, Abcam), as per the manufacturer’s protocol.

### NADP/NADPH estimation

For estimating NADP/NADPH ratio, a commercially available kit (ab65349, Abcam) was utilized, as per the manufacturer’s protocol.

### Western blotting

Whole-cell protein lysates (30 µg), separated on sodium dodecyl sulfate-polyacrylamide gels, were assayed for protein expression, utilizing specific antibodies (G6PD, ab993 and ab76598, Abcam; p53, 554166, BD Biosciences) and chemiluminescent detection (ECL kit, Sigma Aldrich). β-Actin (A2228, Sigma) served as an internal control for whole-cell extracts.

### Experimental animals and ethics statement

Male Sprague Dawley rats weighing 220–250 g were used for the study. The animals were maintained under standard conditions in animal house facility of the institute and were exposed to a 12:12 h light–dark cycle. They were given pellet diet and water ad libitum. In all, 3–4 rats were housed per cage during all experimental procedures. The study design was approved by the standing ‘‘Institute Animal Ethics Committee’’, DIPAS, DRDO and the experiments were conducted in compliance with the recommended guidelines of ‘‘Committee for the Purpose of Control and Supervision of Experiments on Animals (CPCSEA)’’, Government of India.

### Drug administration

The animals were randomly assigned to four groups namely; control, isoproterenol (ISO), isoproterenol_NaHS (ISO_NaHS), and NaHS. ISO groups received isoproterenol at a dose of 5 mg per kg of body weight (in saline, 0.9 % NaCl), once daily by the subcutaneous route. For H_2_S donor groups, the animals received daily intraperitoneal (i.p.) injection of NaHS (Cayman Chemicals) at 4 mg per kg of body weight per day for 12 days, prior to isoproterenol challenge. NaHS (2 mg/ml) was prepared fresh in 0.15 M phosphate-buffered saline (PBS), pH 7.4 before each injection^61^. The control groups received sham injections with an equal volume of 0.15 M PBS. Drug administration was continued for the entire period of adrenergic stimulation.

For G6PD inhibitor treatment, the animals received intraperitoneal (i.p.) injection of 6-aminonicotinamide (6-AN, Sigma Aldrich), prepared in 4% DMSO, at 1 mg per kg of body weight, after every 5 days. Beginning with the first dose of NaHS, four uniformly spaced doses of 6-AN were administered to the animals during entire experimental duration.

### Sulfide estimation in heart tissues

For estimation of total free sulfide, modified zinc precipitation assay^63^ was employed as per protocol described by us previously^61^. The standard curve in the assay was prepared utilizing known concentrations of NaHS.

### Assessment of general parameters of animals

The final body weight (BW, measured in g), tail length (TL, measured in cm) along with heart weight (HW, measured in mg) were recorded on the day of sacrifice and used to calculate the heart-to-body weight (HW/BW) or heart weight-to-tail length (HW/TL) ratios.

### Morphometry

Heart tissues were fixed in 4% paraformaldehyde (PFA) after washing with ice cold PBS (pH 7.4). The auricles were removed and transverse ventricular sections of 2 mm thickness prepared (equidistant from the base of heart). The sections were imaged (with appropriate scale) and subsequently, analyzed (to estimate left ventricular-free-wall thickness and left ventricular-cavity area) utilizing Image J, NIH. The wall thickness was measured for three distinct regions of the chamber (similar regions in all the groups/animals).

### Histological assessment

Heart tissues were fixed in 4% PFA, dehydrated through series of increasing ethanol concentration, cleared in xylene, and paraffin-embedded. Transverse sections from mid-region of heart were cut serially at 5 μm thickness. The sections were de-paraffinized and hydrated prior to staining. For Picro-sirius red (PSR80) staining, sections were incubated for 60 min in 0.1% Sirius red (Sigma) dissolved in saturated picric acid, rinsed in acidified water, dehydrated in ethanol, and cleared in xylene. For Masson Trichrome (MT) staining, a commercially available kit (HT15, Sigma Aldrich) was used, as per manufacturer’s protocol. Images of stained sections were acquired utilizing optical microscope (BX51, Olympus Corporation, Tokyo, Japan or AE31 Motic, China). A minimum of four independent fields (within a minimum of three random sections) per group was imaged and analyzed.

### Transmission electron microscopy

The samples were prepared as per protocol described us previously^61^ and images acquired using transmission electron microscope (Technai G2, The Netherlands).

### Statistical analysis

The statistical significance of data within multiple groups of a specific experiment was evaluated by one-way analysis of variance (*P < 0.05, **P < 0.01, ***P < 0.001). Mean ± SE was calculated for data sets with at least three independent observations and represented using bar graphs.

## Electronic supplementary material

Supplementary Figures

Supplementary Table 1

Supplementary Table 2
