# Supplementary material for: Glucose-6-phosphate dehydrogenase is critical for suppression of cardiac hypertrophy by H2S
Source: Cell Death Discov. 2018 Feb 1;4:6. doi: 10.1038/s41420-017-0010-9 (PMC5841415; doi:10.1038/s41420-017-0010-9)

LEGENDS TO SUPPLEMENTARY FIGURES

Supplementary Figure 1A-J: *KEGG pathways significantly enriched from differentially expressed genes in H9c2 cells treated with NaHS.* KEGG pathways (top 10), extracted from DAVID Bioinformatics resource, significantly enriched from differentially expressed genes. The relative expression (w.r.t control) for specific genes, within these pathways, is indicated by color; Red – Up-regulated, Green – down-regulated, Yellow – Group consisting of both up-regulated and down-regulated genes, Blue – Gene symbol.

Supplementary Figure 2: *Evaluation of effects of G6PD inhibitor, 6-AN, on heart tissues of animals.* The animals were administered 4 uniformly spaced doses of 6-AN (1 mg/kg b.w., after every 5 days, i.p) and various parameters (related to heart) were studied. **A)** Representative gross heart images from the indicated groups (n=5) **B-C)** Representative bright field micrographs of Haematoxylin and Eosin (H&E, panel **B**) & Picro-Sirius Red (PSR 80, panel **C**) stained sections of heart tissues from the indicated groups. **D-F)** Bar graphs (Mean ± S.E.) depicting heart weight (HW):body weight (BW) ratios (in mg/g), heart weight (HW):tail length (TL) ratios (in mg/cm) and left ventricular free wall thickness (in mm) from the indicated groups (n=5).

LEGENDS TO SUPPLEMENTARY TABLES

Supplementary Table 1: *List of differentially expressed genes (compared to Control), as inferred from transcriptome analysis of H9c2 cells treated with 400 µM NaHS for 6 hrs.* Accession Number, Gene Symbol and respective Fold change (Log_2) are shown along with other details as indicated.

Supplementary Table 2: *List of KEGG pathways significantly enriched from differentially expressed genes in H9c2 cells treated with 400 µM NaHS for 6 hrs.* The differentially expressed genes were subjected to analysis employing DAVID Bioinformatics resource and the list of KEGG pathways significantly enriched from this data set along with their respective KEGG ID, *p*-Value, Accession No., Gene Symbol, Gene Name, Fold change (Log_2 values) and function is represented.

Supplementary Figure 1A (Steroid biosynthesis)

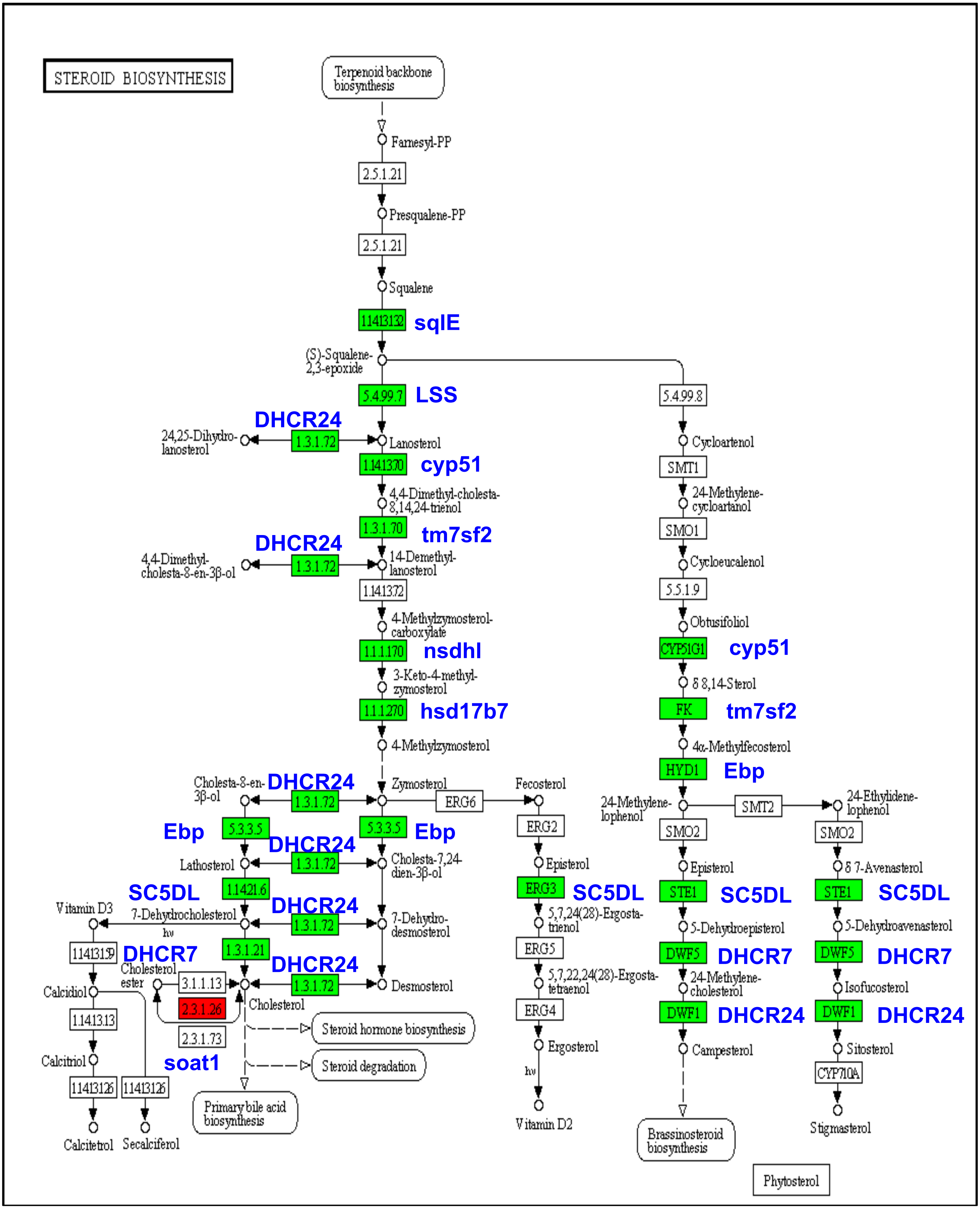

Supplementary Figure 1B (Terpenoid backbone biosynthesis)

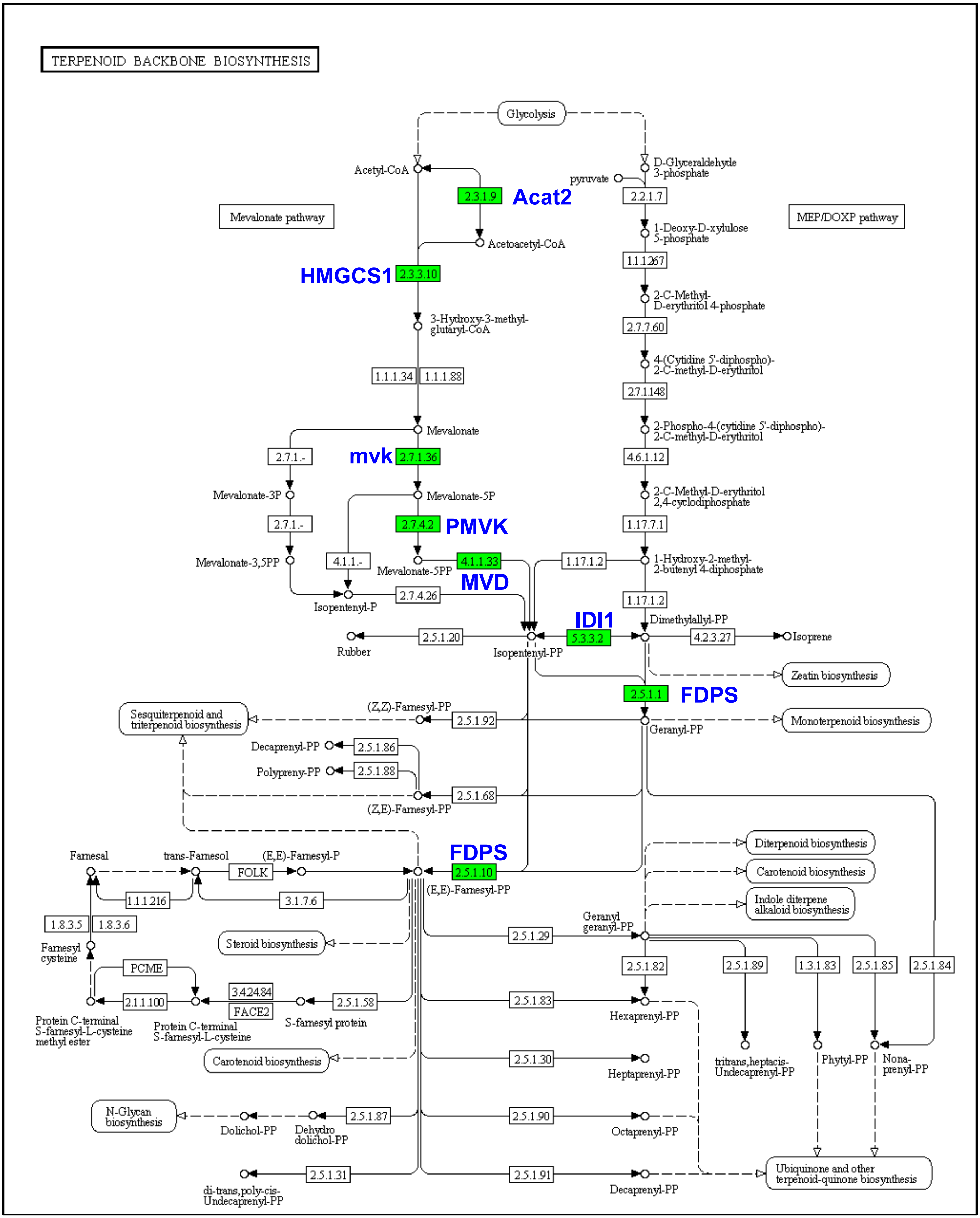

Supplementary Figure 1C (Pentose phosphate pathway)

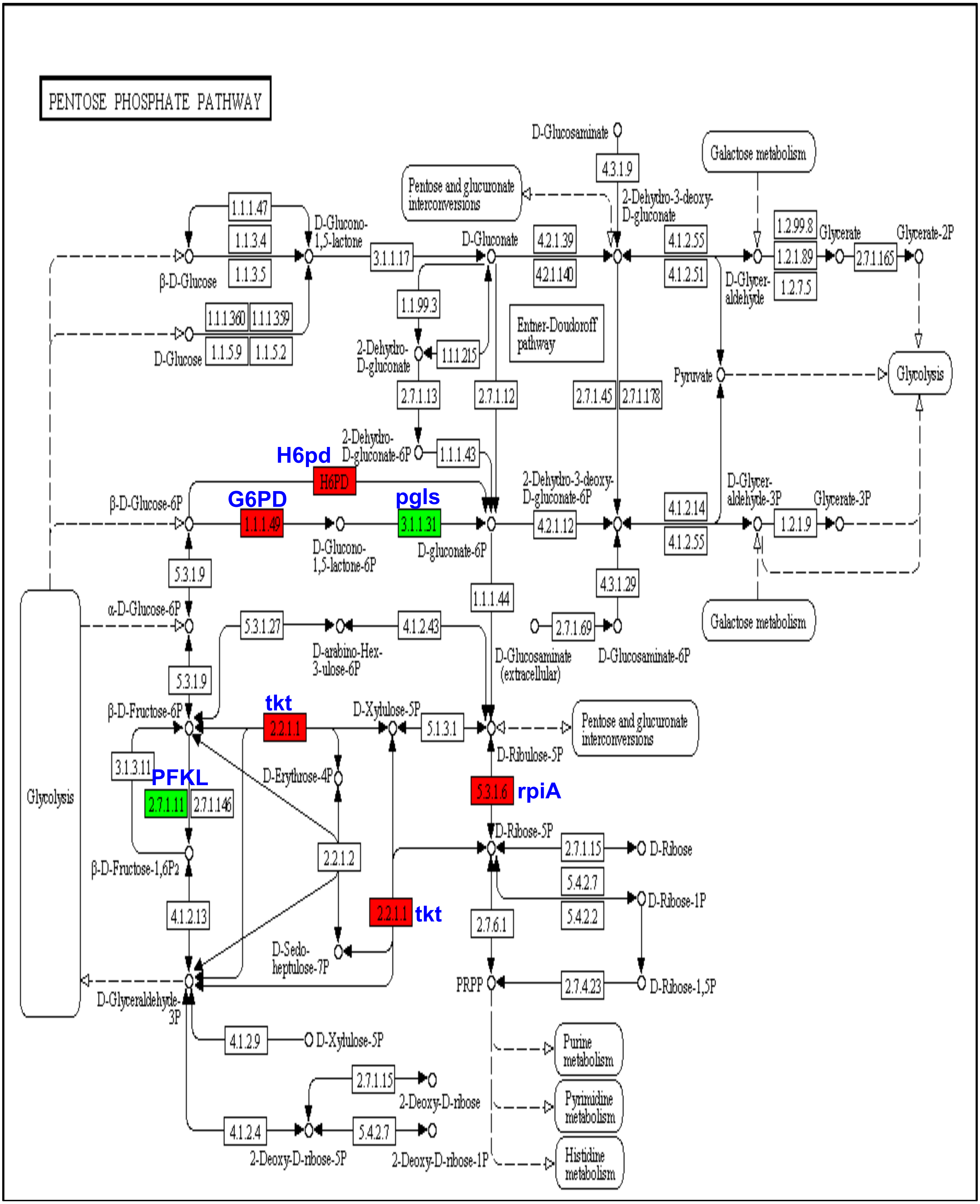

Supplementary Figure 1D (Glutathione metabolism)

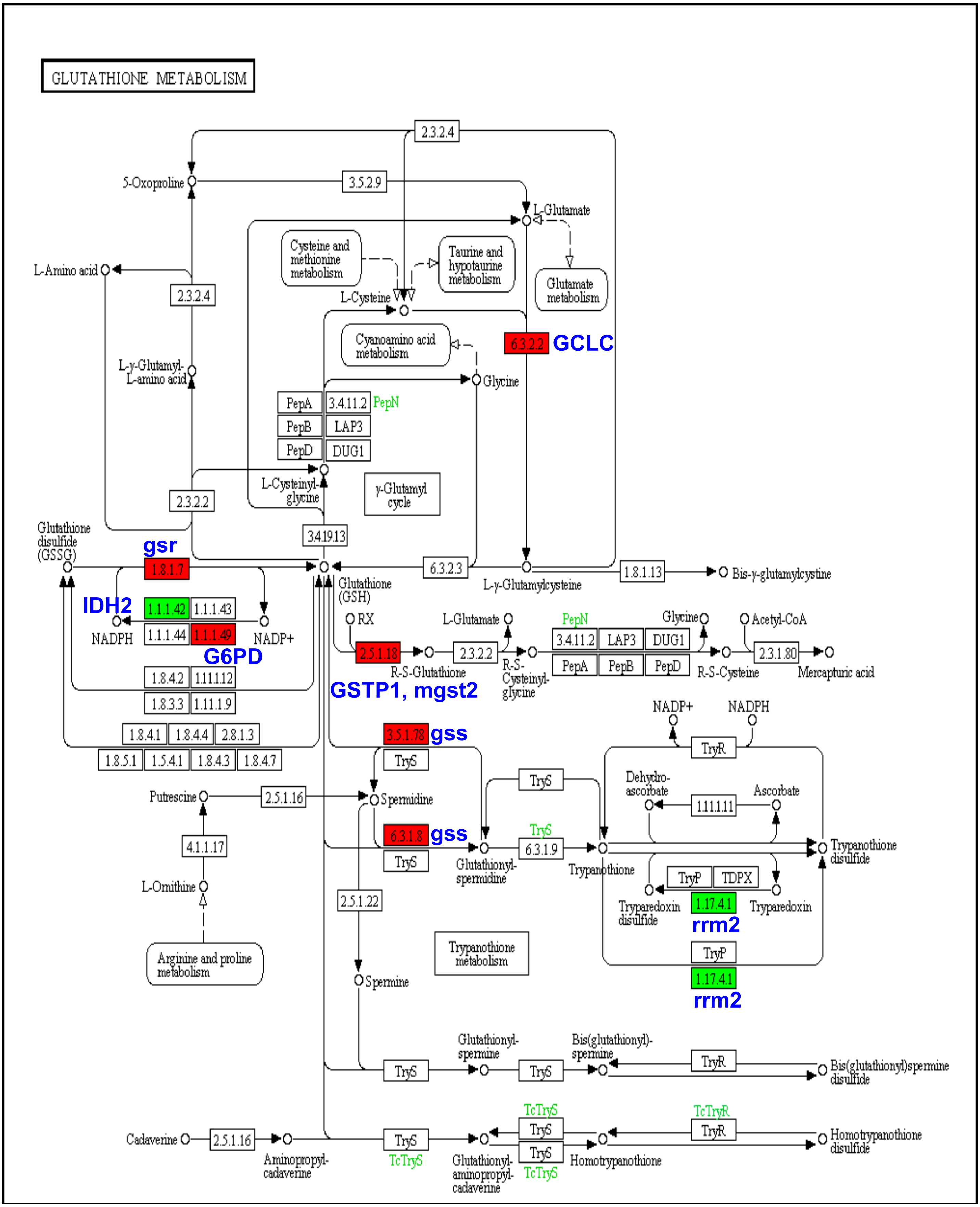

Supplementary Figure 1E (Small cell lung cancer)

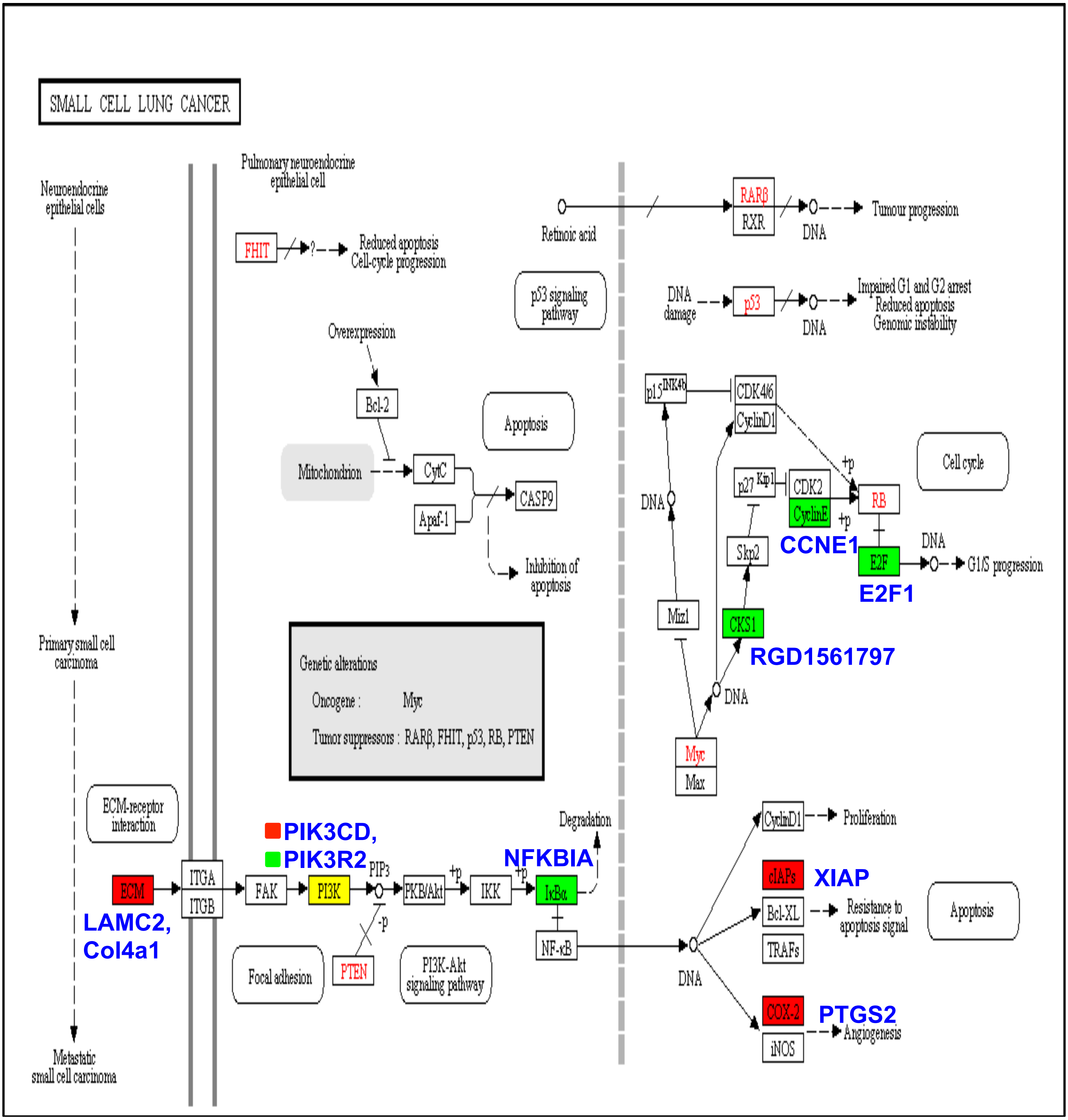

Supplementary Figure 1F (Focal adhesion)

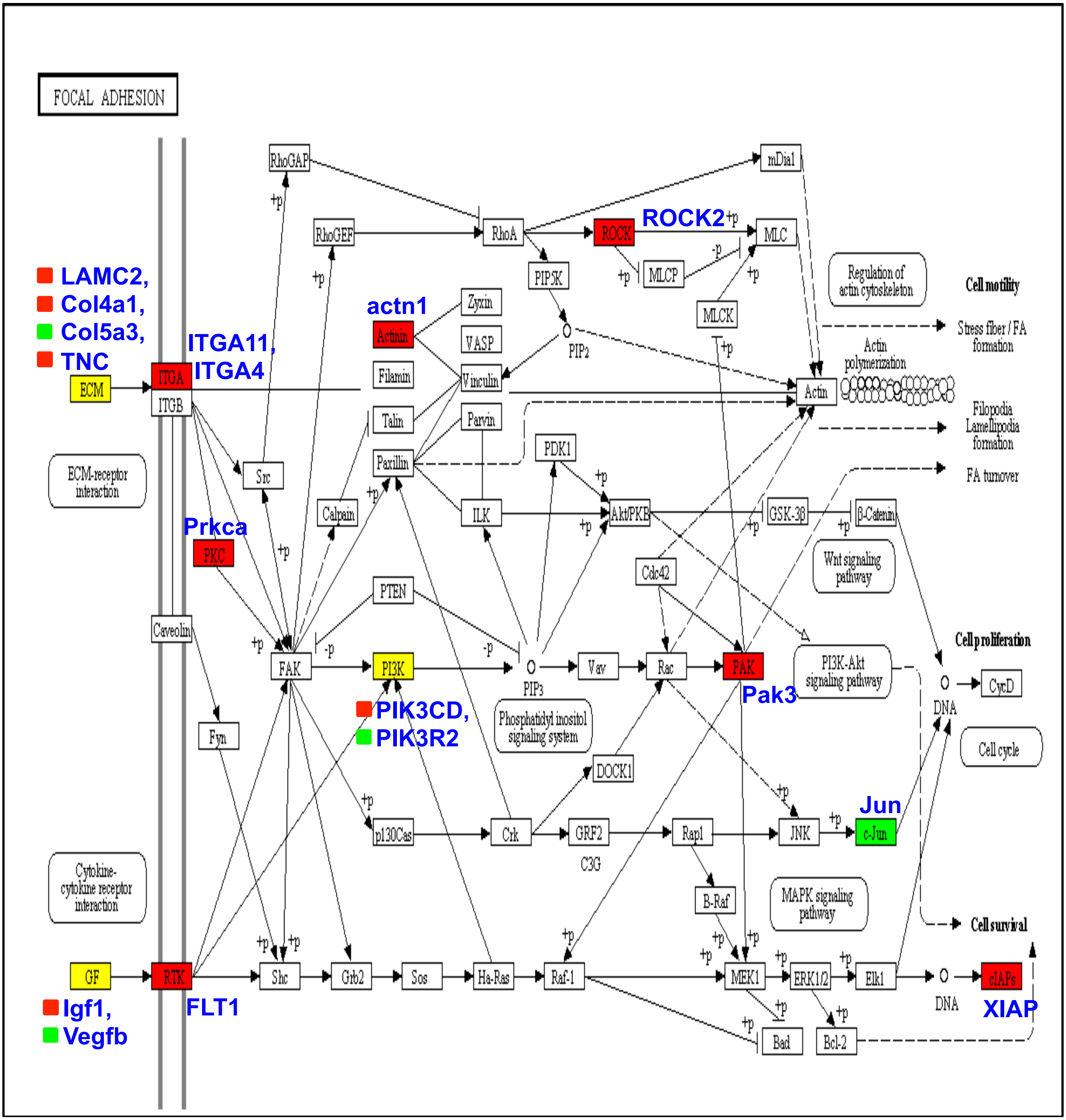

Supplementary Figure 1G (Nicotinate and nicotinamide metabolism)

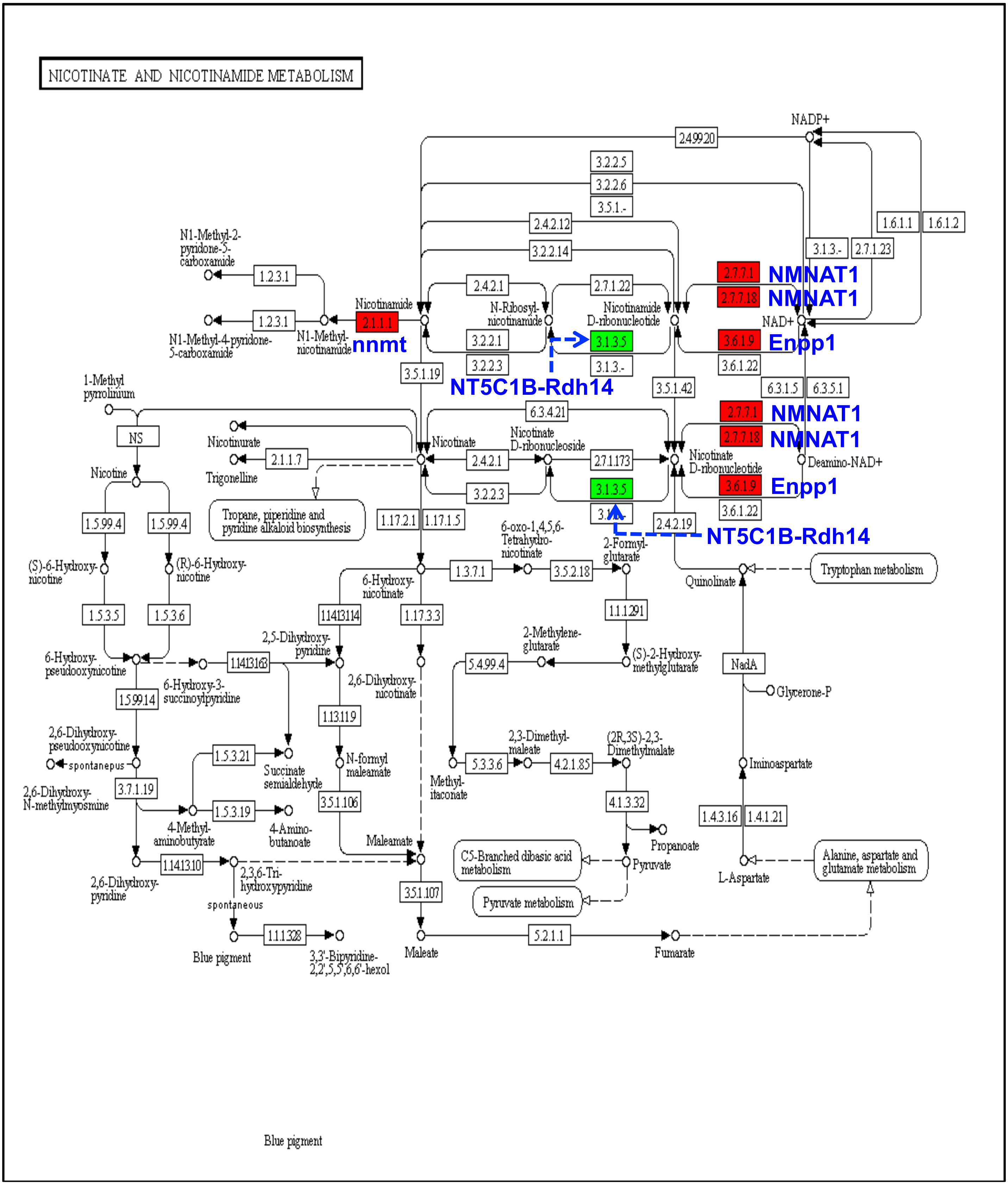

Supplementary Figure 1H (Axon guidance)

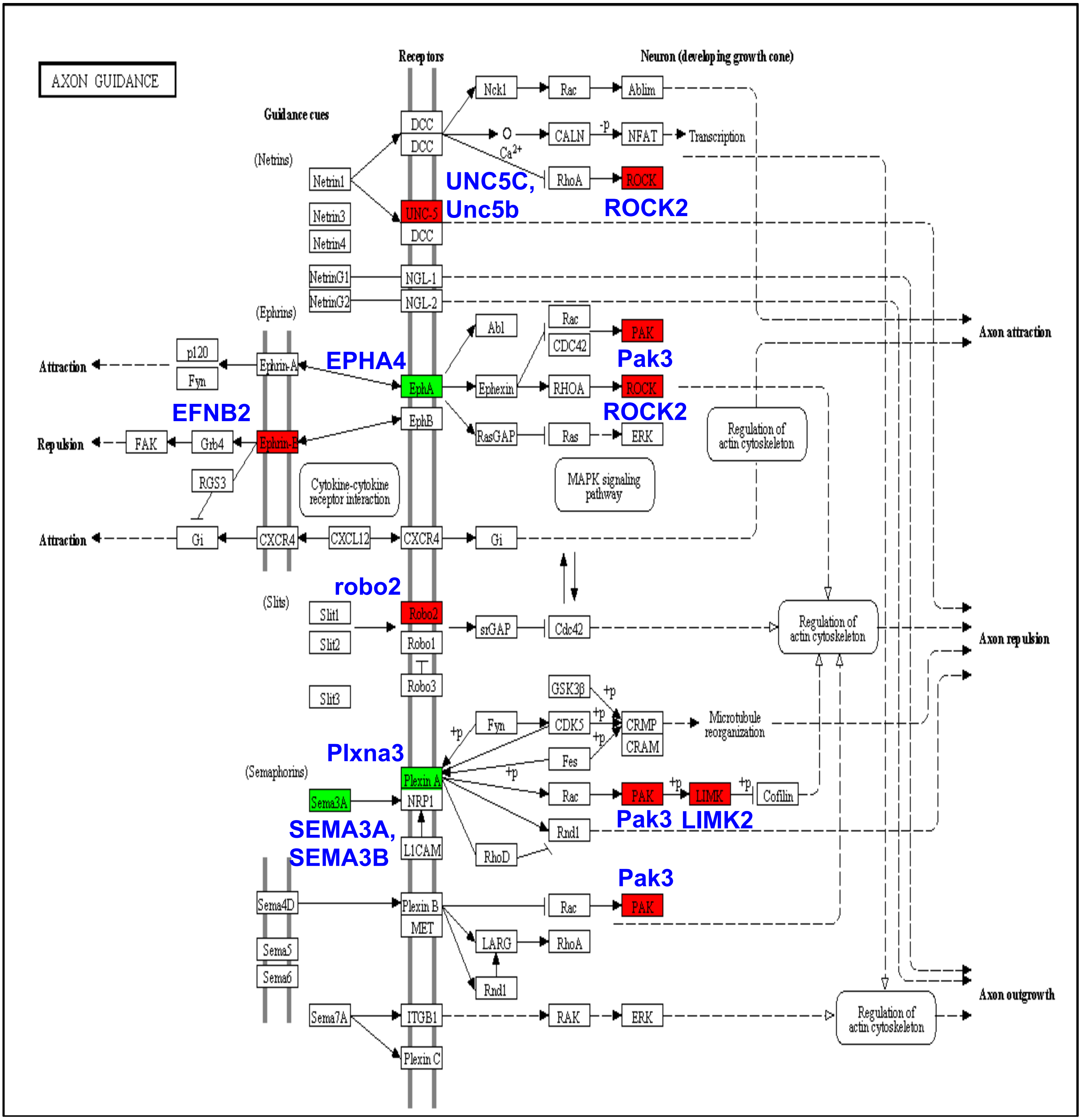

Supplementary Figure 1I (Pyrimidine metabolism)

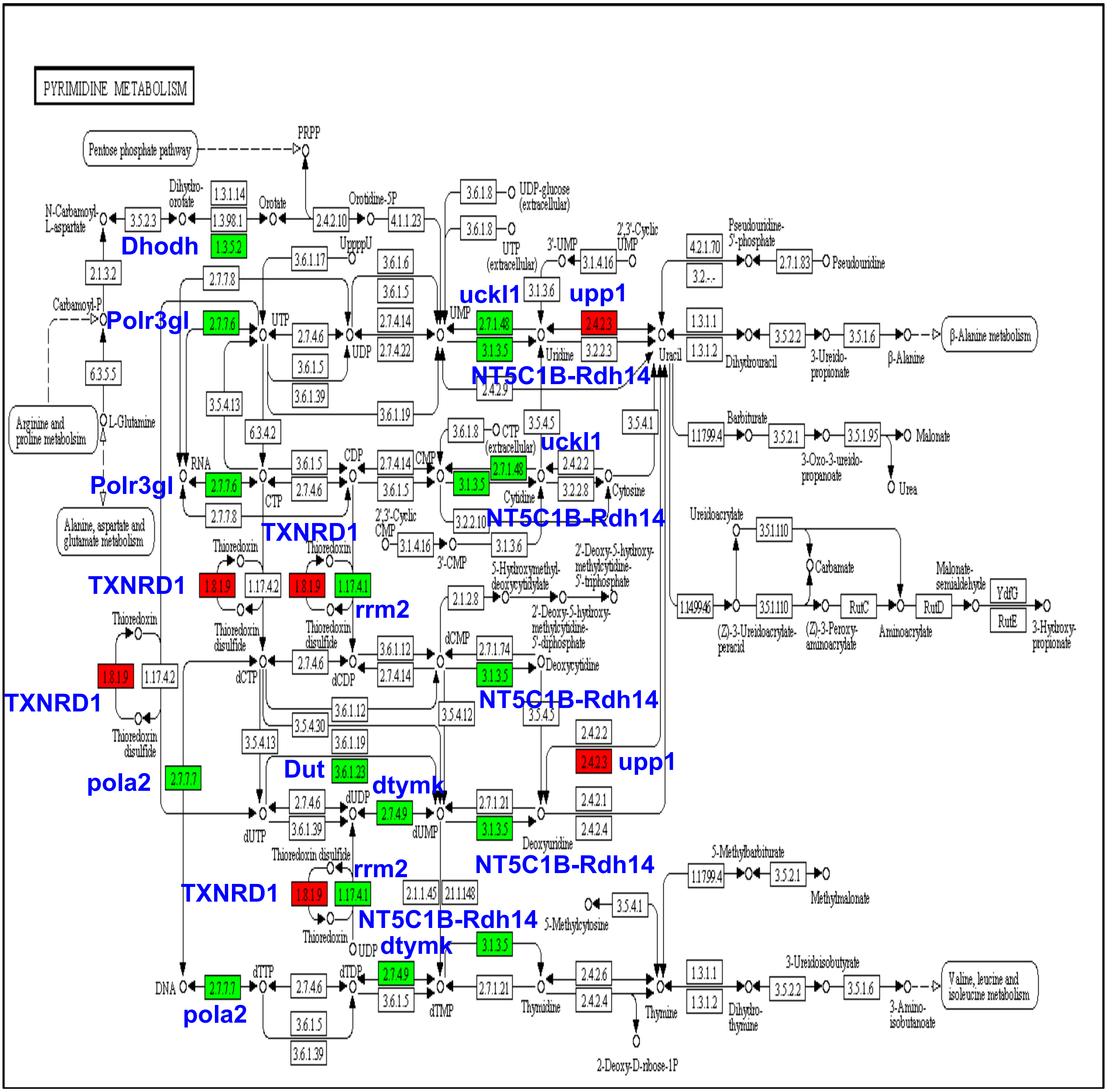

Supplementary Figure 1J (p53 signaling pathway)

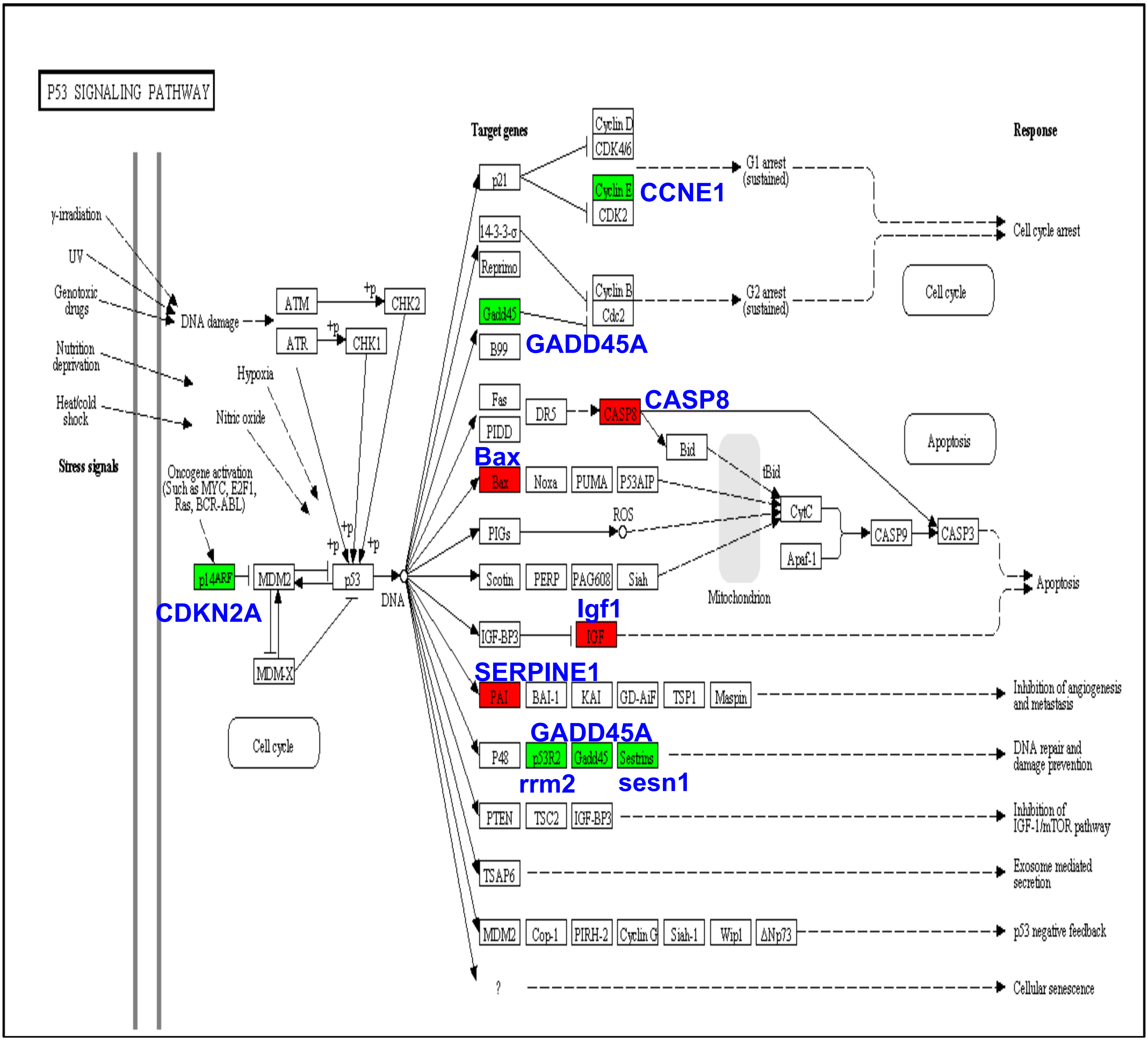

Supplementary Figure 2

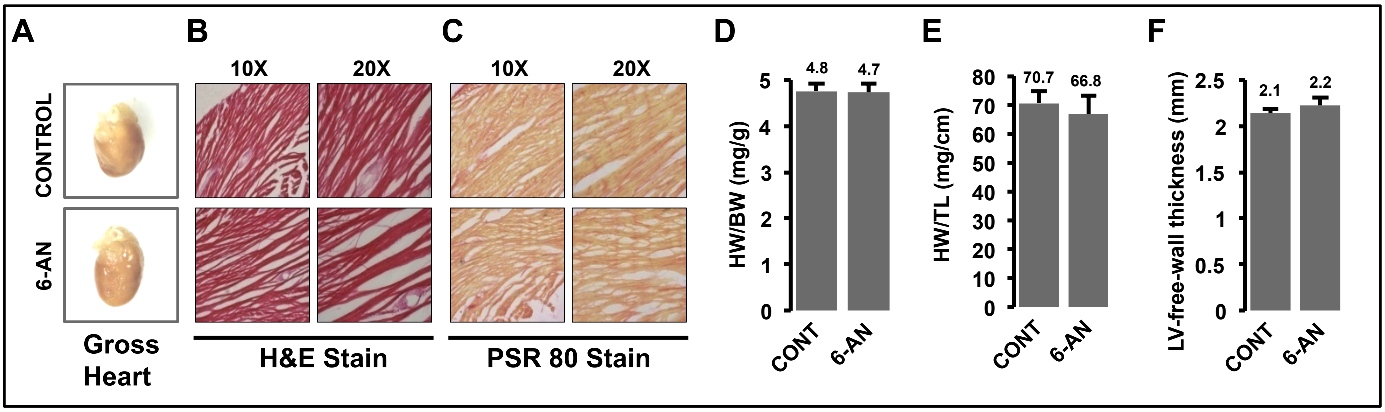

Supplement: Supplementary file 1 — Supplementary Figures [file 41420_2017_10_MOESM1_ESM.pdf]
