# Supplementary material for: Glucose-6-phosphate dehydrogenase is critical for suppression of cardiac hypertrophy by H2S
Source: Cell Death Discov. 2018 Feb 1;4:6. doi: 10.1038/s41420-017-0010-9 (PMC5841415; doi:10.1038/s41420-017-0010-9)
Supplement: Supplementary file 3 — Supplementary Table 2 [file 41420_2017_10_MOESM3_ESM.pdf]

Supplementary Table 2

S.No.	KEGG Pathway	KEGG ID	P-Value	Accesion No.	Gene Symbol	Gene Name	Fold Change	Possible Function
**1**	**Steroid biosynthesis**	**rno00100**	3.70E-09					
				NM_031118	soat1	sterol O-acyltransferase 1	0.465949785	Formation of fatty acid-cholesterol esters. Role in lipoprotein assembly and dietary cholesterol absorption
				NM_001080148	DHCR24	24-dehydrocholesterol reductase	-0.421451719	Reduction of delta-24 double bond of sterol intermediates. Protects cells from oxidative stress and amyloid-beta peptide-induced apoptosis
				NM_012941	cyp51	cytochrome P450, subfamily 51	-0.607723294	Catalyzes C14-demethylation of lanosterol
				NM_001009399	nsdhl	NAD(P) dependent steroid dehydrogenase-like	-0.604989625	Role in cholesterol biosynthesis with 3-beta-hydroxy-delta5-steroid dehydrogenase and sterol-4-alpha-carboxylate 3-dehydrogenase activity
				NM_022389	DHCR7	7-dehydrocholesterol reductase	-0.753145419	Cholesterol production by reduction of C7-C8 double bond of 7-dehydrocholesterol (7-DHC)
				NM_017136	sqlE	squalene epoxidase	-0.547033944	Rate limiting enzyme catalyzing first oxygenation step in sterol biosynthesis
				NM_057137	Ebp	emopamil binding protein (sterol isomerase)	-1.16095	Conversion of Delta(8)-sterols to their corresponding Delta(7)-isomers
				NM_053642	SC5DL	sterol-C5-desaturase (ERG3 delta-5-desaturase homolog, S. cerevisiae)-like	-0.594657641	Catalyzes a dehydrogenation to introduce C5-6 double bond into lathosterol
				NM_017235	hsd17b7	hydroxysteroid (17-beta) dehydrogenase 7	-0.693201645	Reduction of the keto group on the C-3 of sterols
				NM_001013071	tm7sf2	transmembrane 7 superfamily member 2	-1.720922849	Involved in conversion of lanosterol to cholesterol
				NM_031049	LSS	lanosterol synthase (2,3-oxidosqualene-lanosterol cyclase)	-0.855996335	Catalyzes the cyclization of (S)-2,3 oxidosqualene to lanosterol
**2**	**Terpenoid backbone biosynthesis**	**rno00900**	8.20E-06					
				NM_017268	HMGCS1	3-hydroxy-3-methylglutaryl-Coenzyme A synthase 1 (soluble)	-0.416646752	Condensation of acetyl-CoA with acetoacetyl-CoA to form HMG-CoA
				NM_001006995	Acat2	similar to Ab2-076; acetyl-Coenzyme A acetyltransferase 2	-0.625401113	Involved in lipid metabolism. Encodes for cytosolic acetoacetyl-CoA thiolase
				NM_031062	MVD	mevalonate (diphospho) decarboxylase	-0.980940427	Performs the first committed step in isoprene biosynthesis

				NM_031063	mvk	mevalonate kinase	-0.655755683	Possible regulatory site in cholesterol biosynthetic pathway
				NM_031840	FDPS	farnesyl diphosphate synthase (farnesyl pyrophosphate synthetase, dimethylallyltransferase, geranyltranstransferase); similar to testis-specific farnesyl pyrophosphate synthetase	-0.416103167	Catalyzes the formation of farnesyl diphosphate (FPP)
				NM_053539	IDI1	isopentenyl-diphosphate delta isomerase 1	-0.718316936	Catalyzes the 1,3-allylic rearrangement of isopentenyl (IPP) to dimethylallyl diphosphate (DMAPP)
				NM_001008352	PMVK	phosphomevalonate kinase	-0.692957855	Catalyzes conversion of mevalonate 5-phosphate into mevalonate 5-diphosphate
3	**Pentose phosphate pathway**	**rno00030**	2.10E-03					
				NM_001106066	pgls	6-phosphogluconolactonase	-0.955967017	Hydrolysis of 6-phosphogluconolactone to 6-phosphogluconate
				NM_001108632	rpiA	ribose 5-phosphate isomerase A	0.79845852	Catalyzes the reversible conversion between ribose-5-phosphate and ribulose-5-phosphate
				NM_001106698	H6pd	hexose-6-phosphate dehydrogenase (glucose 1-dehydrogenase)	0.952752316	Oxidation of glucose-6-phosphate and glucose, as well as other hexose-6-phosphates
				NM_013190	PFKL	phosphofructokinase, liver	-0.569591121	Catalyzes phosphorylation of fructose-6-phosphate (F6P) by ATP to generate fructose-1,6-bisphosphate (FBP) and ADP
				NM_022592	tkt	transketolase	0.454168534	Catalyzes transfer of 2-C ketol group from a ketose donor to an aldose acceptor
				NM_017006	G6PD	glucose-6-phosphate dehydrogenase	0.508504519	Provides reducing power (NADPH) and pentose phosphates for fatty acid and nucleic acid synthesis
4	**Glutathione metabolism**	**rno00480**	3.20E-03					
				NM_012815	GCLC	glutamate-cysteine ligase, catalytic subunit	0.630291892	First rate-limiting enzyme of glutathione synthesis
				NM_012962	gss	glutathione synthetase	0.494542328	Catalyzes ATP-dependent conversion of gamma-L-glutamyl-L-cysteine to glutathione

				NM_001025740	rrm2	similar to M2 ribonucleotide reductase; ribonucleotide reductase M2	-0.488456683	Provides precursors necessary for DNA synthesis. Catalyzes biosynthesis of deoxyribonucleotides from corresponding ribonucleotides
				NM_012577	GSTP1	glutathione-S-transferase, pi 1	0.733493476	Conjugation of reduced glutathione to various exogenous and endogenous hydrophobic electrophiles. Negative regulation of CDK5 activity
				NM_001014161	IDH2	isocitrate dehydrogenase 2 (NADP+), mitochondrial	-0.398972684	Role in intermediary metabolism and energy production. Associates with pyruvate dehydrogenase complex
				NM_001106430	mgst2	microsomal glutathione S-transferase 2	0.654670073	Catalyzes production of LTC4 from LTA4 and reduced glutathione as well as conjugation of 1-chloro-2,4-dinitrobenzene with reduced glutathione
				NM_053906	gsr	glutathione reductase	0.833119904	Maintains high levels of reduced glutathione in the cytosol
				NM_017006	G6PD	glucose-6-phosphate dehydrogenase	0.508504519	Provides reducing power (NADPH) and pentose phosphates for fatty acid and nucleic acid synthesis
5	**Small cell lung cancer**	**rno05222**	5.20E-03					
				NM_001108978	PIK3CD	phosphatidylinositol 3-kinase catalytic delta polypeptide	0.541557918	Phosphorylates PftdIns(4,5)P2 (Phosphatidylinositol 4,5-bisphosphate) to generate phosphatidylinositol 3,4,5-trisphosphate (PIP3)
				NM_001100821	CCNE1	cyclin E1	-0.776779284	Controls the cell cycle at the G1/S (start) transition
				NM_001100640	LAMC2	laminin, gamma 2	1.632414741	Mediates the attachment, migration and organization of cells into tissues during development
				NM_001100778	E2F1	E2F transcription factor 1	-0.532372934	Controls cell cycle and action of tumor suppressor proteins
				NM_017232	PTGS2	prostaglandin-endoperoxide synthase 2	0.610592366	Involved in prostaglandin biosynthesis. Acts as dioxygenase as well as peroxidase
				NM_022185	PIK3R2	phosphoinositide-3-kinase, regulatory subunit 2 (beta)	-0.702741771	Binds to activated (phosphorylated) protein-tyrosine kinases and acts as an adapter mediating association of p110 catalytic unit to the plasma membrane
				NM_001135749	RGD1561797	similar to Cyclin-dependent kinases regulatory subunit 1 (CKS-1) (Sid 1334); RGD1561797	-0.60431359	Binds to catalytic subunit of the cyclin dependent kinases and is important for their biological function
				NM_001105720	NFKBIA	nuclear factor of kappa light polypeptide gene enhancer in B-cells inhibitor, alpha	-0.82098197	Interacts with REL dimers to inhibit NF-kappa-B/REL complexes involved in inflammatory responses

				NM_022231	XIAP	X-linked inhibitor of apoptosis	0.473212763	Regulates caspases and apoptosis as well as inflammatory signaling and immunity, copper homeostasis, mitogenic kinase signaling, cell proliferation, cell invasion and metastasis
				NM_001135009	Col4a1	collagen, type IV, alpha 1	0.572544492	Encodes for the major structural component of glomerular basement membranes (GBM), forming a meshwork together with laminins, proteoglycans and entactin/nidogen
6	**Focal adhesion**	**rno04510**	9.90E-03					
				NM_019210	Pak3	p21 protein (Cdc42/Rac)-activated kinase 3	0.637159864	Serine/threonine protein kinase that plays role in different signaling pathways including cytoskeleton regulation, cell migration, or cell cycle regulation.
				NM_013022	ROCK2	Rho-associated coiled-coil containing protein kinase 2	0.378341479	Serine/threonine kinase that regulates cytokinesis, smooth muscle contraction, formation of actin stress fibers and focal adhesions and c-fos serum activation
				NM_053861	TNC	tenascin C	0.997547905	Guides migrating neurons as well as axons during development, synaptic plasticity as well as neuronal regeneration. Ligand for integrins alpha-8/beta-1, alpha-9/beta-1, alpha-V/beta-3 and alpha-V/beta-6
				NM_019306	FLT1	fms-related tyrosine kinase 1	1.198276264	Acts as cell-surface receptor for VEGFA, VEGFB and PGF. Role in development of embryonic vasculature, regulation of angiogenesis, cell survival, cell migration, macrophage function, chemotaxis and cancer cell invasion
				NM_001108978	PIK3CD	phosphatidylinositol 3-kinase catalytic delta polypeptide	0.541557918	Phosphorylates PftdIns(4,5)P2 (Phosphatidylinositol 4,5-bisphosphate) to generate phosphatidylinositol 3,4,5-trisphosphate (PIP3)
				NM_001100640	LAMC2	laminin, gamma 2	1.632414741	Mediates the attachment, migration and organization of cells into tissues during development
				NM_001105713	Prkca	protein kinase C, alpha	0.620431002	Phosphorylates variety of protein targets and are known to be involved in diverse cellular signaling pathways
				NM_022185	PIK3R2	phosphoinositide-3-kinase, regulatory subunit 2 (beta)	-0.702741771	Binds to activated (phosphorylated) protein-tyrosine kinases and acts as an adapter mediating association of p110 catalytic unit to the plasma membrane
				NM_021835	Jun	Jun oncogene	-0.732293215	Encodes for transcription factor that recognizes and binds to the enhancer heptamer motif 5'-TGA[CG]TCA-3'
				NM_001108156	ITGA11	integrin, alpha 11	0.414115273	Acts as a receptor for collagen
				NM_001135009	Col4a1	collagen, type IV, alpha 1	0.572544492	Encodes for the major structural component of glomerular basement membranes (GBM), forming a meshwork together with laminins, proteoglycans and entactin/nidogen
				NM_021760	Col5a3	collagen, type V, alpha 3	-0.522074289	Minor connective tissue component of nearly ubiquitous distribution. Binds to DNA, heparan sulfate, thrombospondin, heparin and insulin
				NM_031005	actn1	actinin, alpha 1	0.719618382	F-actin cross-linking protein that anchors actin to a variety of intracellular structures
				NM_178866	Igf1	insulin-like growth factor 1	1.91823	Similar to insulin in function and structure. Mediates growth and development
				NM_001082477	Igf1	insulin-like growth factor 1	1.91823	Similar to insulin in function and structure. Mediates growth and development

				NM_053549	Vegfb	vascular endothelial growth factor B; DnaJ (Hsp40) homolog, subfamily C, member 4	-0.803992587	Growth factor for endothelial cells
				NM_001082479	Igf1	insulin-like growth factor 1	1.91823	Similar to insulin in function and structure. Mediates growth and development
				NM_001107737	ITGA4	integrin alpha 4	1.189945545	Acts as receptor for fibronectin and VCAM1
				NM_001082478	Igf1	insulin-like growth factor 1	1.91823	Similar to insulin in function and structure. Mediates growth and development
				NM_022231	XIAP	X-linked inhibitor of apoptosis	0.473212763	Regulates caspases and apoptosis as well as inflammatory signaling and immunity, copper homeostasis, mitogenic kinase signaling, cell proliferation, cell invasion and metastasis
7	**Nicotinate and nicotinamide metabolism**	**rno00760**	1.20E-02					
				NM_001106031	NP	nucleoside phosphorylase	0.588619474	Catalyzes phosphorolytic breakdown of the N-glycosidic bond in the beta-(deoxy)ribonucleoside molecules
				NM_001109276	Rdh14	retinol dehydrogenase 14 (all-trans/9-cis/11-cis); 5'-nucleotidase, cytosolic IB	-1.04797	Oxidoreductive catalytic activity towards retinoids. Most efficient as an NADPH-dependent retinal reductase
				NM_053535	Enpp1	ectonucleotide pyrophosphatase/phosphodiesterase 1	0.805258232	Regulates pyrophosphate levels by generating PPi. Functions in bone mineralization and soft tissue calcification
				NM_001011982	NT5C1B	retinol dehydrogenase 14 (all-trans/9-cis/11-cis); 5'-nucleotidase, cytosolic IB	-0.306125265	Dephosphorylates the 5' and 2'(3')-phosphates of deoxyribonucleotides. Helps to regulate adenosine levels
				NM_001106819	nnmt	nicotinamide N-methyltransferase	0.829597001	Catalyzes N-methylation of nicotinamide and other pyridines to form pyridinium ions
				NM_001037556	NMNAT1	nicotinamide nucleotide adenylyltransferase 1	1.207687092	Catalyzes formation of NAD(+) from nicotinamide mononucleotide (NMN) and ATP
8	**Axon guidance**	rno04360	2.80E-02	NM_001162411	EPHA4	Eph receptor A4	-0.691034	Receptor tyrosine kinase which binds membrane-bound ephrin family ligands residing on adjacent cells, leading to contact-dependent bidirectional signaling into neighboring cells
				NM_024135	LIMK2	LIM domain kinase 2	0.689591	Shows serine/threonine-specific phosphorylation of myelin basic protein and histone (MBP) *in vitro*

				NM_013022	ROCK2	Rho-associated coiled-coil containing protein kinase 2	0.378343	Serine/threonine kinase that regulates cytokinesis, smooth muscle contraction, formation of actin stress fibers and focal adhesions and c-fos serum activation
				NM_001107328	EFNB2	ephrin B2	0.673199	Cell surface transmembrane ligand for Eph receptors, which are crucial for migration, repulsion and adhesion during neuronal, vascular and epithelial development
				NM_019210	Pak3	p21 protein (Cdc42/Rac)-activated kinase 3	0.637158	Serine/threonine protein kinase that plays role in different signaling pathways including cytoskeleton regulation, cell migration, or cell cycle regulation
				NM_001107581	Plxna3	plexin A3	-0.545184	Coreceptor for SEMA3A and SEMA3F. Necessary for signaling by Class 3 semaphorins and subsequent remodeling of cytoskeleton
				NM_032106	robo2	roundabout homolog 2 (Drosophila)	0.657547	Receptor for SLIT2 and probably, SLIT1, which are thought to act as molecular guidance cues in cellular migration
				NM_017310	SEMA3A	sema domain, immunoglobulin domain (Ig), short basic domain, secreted, (semaphorin) 3A	-0.881903	Involved in the development of olfactory system and in neuronal control of puberty
				NM_001079942	SEMA3B	sema domain, immunoglobulin domain (Ig), short basic domain, secreted, (semaphorin) 3B	-0.538916	Inhibits axonal extension by providing local signals to specify territories inaccessible for growing axons (By similarity)
				NM_022207	Unc5b	unc-5 homolog B (C. elegans)	0.482943	Receptor for netrin required for axon guidance. Mediates axon repulsion of neuronal growth cones in the developing nervous system upon ligand binding
				NM_199407	UNC5C	unc-5 homolog C (C. elegans)	0.922738	Receptor for netrin required for axon guidance. Mediates axon repulsion of neuronal growth cones in the developing nervous system upon ligand binding
9	**Pyrimidine metabolism**	**rno00240**	2.80E-02					
				NM_053592	Dut	deoxyuridine triphosphatase	-0.522103	Produces dUMP and decreases the intracellular concentration of dUTP
				NM_001008553	Dhodh	dihydroorotate dehydrogenase	-1.05522	Catalyzes the conversion of dihydroorotate to orotate with quinone as electron acceptor
				NM_001030025	upp1	uridine phosphorylase 1	1.844797714	Catalyzes reversible phosphorylytic cleavage of uridine and deoxyuridine to uracil and ribose- or deoxyribose-1-phosphate
				NM_001109212	uckl1	uridine-cytidine kinase 1-like 1	-0.604072347	Contributes to UTP accumulation required for blast transformation and proliferation
				NM_001109571	Polr3gl	polymerase (RNA) III (DNA directed) polypeptide G (32kD)-like	-0.903218325	DNA-directed RNA polymerase activity

				NM_001106031	NP	nucleoside phosphorylase	0.588619474	Catalyzes phosphorolytic breakdown of the N-glycosidic bond in the beta-(deoxy)ribonucleoside molecules
				NM_001106925	dtymk	deoxythymidylate kinase (thymidylate kinase)	-0.572196075	Catalyzes the conversion of dTMP to dTDP
				NM_001025740	rrm2	similar to M2 ribonucleotide reductase; ribonucleotide reductase M2	-0.488456683	Provides precursors necessary for DNA synthesis. Catalyzes biosynthesis of deoxyribonucleotides from corresponding ribonucleotides
				NM_053480	pola2	polymerase (DNA directed), alpha 2	-0.650465155	Couples polymerase alpha/primase complex to the cellular replication machinery during DNA replication
				NM_001109276	Rdh14	retinol dehydrogenase 14 (all-trans/9-cis/11-cis); 5'-nucleotidase, cytosolic IB	-1.04797	Oxidoreductive catalytic activity towards retinoids. Most efficient as an NADPH-dependent retinal reductase
				NM_031614	TXNRD1	thioredoxin reductase 1	0.874883124	Catalyzes reduction of thioredoxins as well as other substrates. Plays role in selenium metabolism and protection against oxidative stress
				NM_001011982	NT5C1B	retinol dehydrogenase 14 (all-trans/9-cis/11-cis); 5'-nucleotidase, cytosolic IB	-0.306125265	Dephosphorylates the 5' and 2'(3')-phosphates of deoxyribonucleotides. Helps to regulate adenosine levels
				NM_001040271	Dut	deoxyuridine triphosphatase	-0.522103	Produces dUMP and decreases the intracellular concentration of dUTP
**10**	**p53 signaling pathway**	**rno04115**	4.50E-02					
				NM_031550	CDKN2A	cyclin-dependent kinase inhibitor 2A	-0.914912856	Induces cell cycle arrest in G1 and G2 phases. Acts as tumor suppressor
				NM_001106396	sesn1	sestrin 1	-0.929139144	Involved in reduction of peroxiredoxins. Possible regulator of cellular growth
				NM_001025740	rrm2	similar to M2 ribonucleotide reductase; ribonucleotide reductase M2	-0.488456683	Provides precursors necessary for DNA synthesis. Catalyzes biosynthesis of deoxyribonucleotides from corresponding ribonucleotides
				NM_022277	CASP8	caspase 8	1.004210468	Most upstream protease of the activation cascade of caspases responsible for TNFRSF6/FAS mediated and TNFRSF1A induced cell death
				NM_001100821	CCNE1	cyclin E1	-0.776779284	Controls the cell cycle at the G1/S (start) transition
				NM_178866	Igf1	insulin-like growth factor 1	1.91823	Similar to insulin in function and structure. Mediates growth and development
				NM_017059	Bax	Bcl2-associated X protein	0.716268	Functions as an apoptotic activator

			NM_001082477	Igf1	insulin-like growth factor 1	1.91823	Similar to insulin in function and structure. Mediates growth and development
			NM_001082479	Igf1	insulin-like growth factor 1	1.91823	Similar to insulin in function and structure. Mediates growth and development
			NM_024127	GADD45A	growth arrest and DNA-damage-inducible, alpha	-0.532817707	Mediates activation of p38/JNK pathway via MTK1/MEKK4 kinase
			NM_001082478	Igf1	insulin-like growth factor 1	1.91823	Similar to insulin in function and structure. Mediates growth and development
			NM_012620	SERPINE1	serine (or cysteine) peptidase inhibitor, clade E, member 1	1.843176509	Inhibits tissue plasminogen activator (tPA) and urokinase (uPA)
